# Enhancing accuracy and privacy in speech-based depression detection through speaker disentanglement

**DOI:** 10.1016/j.csl.2023.101605

**Published:** 2023-12-26

**Authors:** Vijay Ravi, Jinhan Wang, Jonathan Flint, Abeer Alwan

**Affiliations:** a Department of Electrical and Computer Engineering, University of California, Los Angeles, CA, 90095, USA; b Department of Psychiatry and Biobehavioral Sciences, University of California, Los Angeles, CA, 90095, USA

**Keywords:** Depression-detection, Speaker-disentanglement, Privacy

## Abstract

Speech signals are valuable biomarkers for assessing an individual’s mental health, including identifying Major Depressive Disorder (MDD) automatically. A frequently used approach in this regard is to employ features related to speaker identity, such as speaker-embeddings. However, over-reliance on speaker identity features in mental health screening systems can compromise patient privacy. Moreover, some aspects of speaker identity may not be relevant for depression detection and could serve as a bias factor that hampers system performance. To overcome these limitations, we propose disentangling speaker-identity information from depression-related information. Specifically, we present four distinct disentanglement methods to achieve this — adversarial speaker identification (SID)-loss maximization (ADV), SID-loss equalization with variance (LEV), SID-loss equalization using Cross-Entropy (LECE) and SID-loss equalization using KL divergence (LEKLD). Our experiments, which incorporated diverse input features and model architectures, have yielded improved F1 scores for MDD detection and voice-privacy attributes, as quantified by Gain in Voice Distinctiveness $\left(G_{V\;D}\right)$ and De-Identification Scores (DeID). On the DAIC-WOZ dataset (English), LECE using ComparE16 features results in the best F1-Scores of 80% which represents the audio-only SOTA depression detection F1-Score along with a $G_{V\;D}$ of −1.1 dB and a DeID of 85%. On the EATD dataset (Mandarin), ADV using raw-audio signal achieves an F1-Score of 72.38% surpassing multi-modal SOTA along with a $G_{V\;D}$ of −0.89 dB dB and a DeID of 51.21%. By reducing the dependence on speaker-identity-related features, our method offers a promising direction for speech-based depression detection that preserves patient privacy.

## 1. Introduction

Major depressive disorder (MDD) is a serious medical illness that adversely affects one’s emotions, thoughts, and behaviors, and in severe cases, can result in suicide. According to James et al. (2018), MDD affects over 264 million people globally and is projected to be the second leading cause of disability by 2030 (Mathers and Loncar, 2006). Mental health problems such as MDD not only have significant economic and healthcare costs but also have a negative impact on the individual, their loved ones, and the community.

Diagnosing MDD currently involves subjective interviews with psychologists and self-reported surveys (Kroenke et al., 2009), which can be affected by the availability of caregivers and patient’s willingness to disclose their symptoms, as well as the societal stigma attached to seeking treatment (Goldman et al., 1999). Therefore, there is an urgent need to develop secure, efficient, accessible, and scalable mental health assessment technologies that can reduce diagnostic inequality and enable early detection of mental health disorders.

While technologies like electroencephalogram (EEG) and Magnetic Resonance Imaging (MRI) have been used to predict mental health states in the past (Liao et al., 2017; Acharya et al., 2015; Mahmood and Ghimire, 2013), they are complex, expensive, and require expert supervision, which limits their scalability. Among others, the human voice has emerged as a promising biomarker for mental health. As an information-rich data source, speech has been shown to effectively capture the mental (Cummins et al., 2015; Ravi et al., 2019) and emotional states (Ramakrishnan, 2012; Park et al., 2018) of the human mind. What is more, speech data can be collected and analyzed non-invasively, without the need for expert supervision, making it a practical and efficient alternative. By extracting representations from speech data, a model can be trained to predict the prevalence of mental health disorders.

Although automatic objective screening mechanisms for MDD have gained popularity in recent years (Bhadra and Kumar, 2022; Safayari and Bolhasani, 2021; Pampouchidou et al., 2017), several challenges remain unresolved. One significant concern for digital healthcare systems, particularly those that involve mental health diagnoses, is privacy. The mental health information of patients is highly personal and confidential, and it is essential to safeguard it to prevent harm such as discrimination, stigma, or social exclusion. Furthermore, individuals may be hesitant to seek mental health care if they feel their information is not secure, which can be counterproductive to the adoption of objective screening systems, leading to untreated conditions and negative health outcomes. Therefore, it is imperative that digital screening systems protect individuals’ privacy to the best possible extent.

From a speech-processing perspective, speaker-related information, also known as speaker-identity features, has been utilized in depression detection using speech-based methods (Dumpala et al., 2022; Ravi et al., 2022a; Egas-López et al., 2022; Dumpala et al., 2023; Liu et al., 2023). However, the use of such features may raise privacy concerns as they can also be used to uniquely identify an individual with the help of automatic speaker identification (SID) (Snyder et al., 2018) and verification models (Ravi et al., 2020). A specific example of privacy threat is the *membership inference attack* (Shokri et al., 2017; Hu et al., 2022), where malicious hackers could compromise the patient’s privacy.

To address these concerns, it is essential for speech-based depression detection models to prioritize preserving individuals’ privacy. Instead of relying on speaker-characteristic information, the focus should be on capturing general patterns that distinguish between depressed and non-depressed populations. By emphasizing the extraction of non-identifying features, the models can contribute to a more privacy-conscious approach in speech-based mental health research.

Regardless of privacy issues, excessive reliance on individual speaker characteristics in depression detection models may introduce dataset biases, leading to poor modeling capability. This bias can cause models to overfit to speakers in the training set, resulting in inaccurate diagnoses for unseen speakers. This raises the question of whether depression detection can be done in a speaker-identity-invariant manner, and whether there are components of speech that characterize a speaker but may not be relevant to their mental health status. These issues have yet to be fully explored in the speech research community.

In this paper, we address these problems by introducing speaker-disentanglement for depression detection, which builds on our previous work (Ravi et al., 2022b). Our approach includes three new methods to address the challenges of adversarial loss maximization proposed in our previous study. We also extend our previous work with additional experiments that incorporate a comprehensive set of input features and backend models. Specifically, the contributions of this paper are threefold:

1. Our preliminary experiments highlight that the inclusion of speaker-related features in MDD detection systems not only compromises privacy but also introduces a bias that can degrade the modeling capabilities of MDD detection.
2. To address the privacy and bias concerns, our proposed approach includes four distinct speaker-disentanglement methods, which encompass three novel loss equalization frameworks involving noise injection with variance, Cross-Entropy, and KL divergence.
3. The performance of our proposed approach surpasses the state-of-the-art (SOTA) methods on two publicly available datasets, one in English (Valstar et al., 2016) (audio-only) and the other in Mandarin (Shen et al., 2022).

The remainder of the paper is organized as follows. In Section 2 we review speech-based depression detection literature. In Section 3, we explain the motivation for this study and introduce the proposed disentanglement methods in Section 4. Section 5 consists of experimental details of models and input features. The results are presented in Section 6 along with a detailed discussion and analysis on model performance. We conclude the paper in Section 7 along with suggestions for future work.

## 2. Literature review

With the advent of digital voice assistants, collecting speech data has become easier, leading to significant attention in research and development of objective speech-based screening systems for Major Depressive Disorder (MDD) (Valstar et al., 2016; Ringeval et al., 2019; Low et al., 2020).

The initial work in this domain focused on analyzing the effect of MDD on human speech. Early studies such as Nilsonne (1988) and Andreasen and Pfohl (1976) demonstrated that MDD is characterized by verbal cues such as monotonic speech, choice of vocabulary, abnormal disfluencies, and other speech-related features. More recent studies have identified discernible differences in the acoustic features of speech between depressed and non-depressed subjects (Cummins et al., 2015; France et al., 2000).

### 2.1. Acoustic features

Previous studies have explored various acoustic features for speech-based depression detection. For instance, in Sanchez et al. (2011), statistics of spectral features such as spectral tilt and formant frequencies were used along with pitch and energy to predict depression. In Yang et al. (2012), vocal prosody features such as switching pauses and pitch were studied for depression severity estimation. Another study (Alghowinem et al., 2013) found that jitter, shimmer, energy, and loudness features were robust for detecting depression in both read and spontaneous speech. While using frame-level features was common, Cummins et al. (2014), Rani (2016) and Di et al. (2021) proposed the use of fixed-length i-vectors for depression detection inspired by speaker-identification literature (Garcia-Romero and Espy-Wilson, 2011). In Afshan et al. (2018), the i-vector representation was extended to voice quality features along with a score-level fusion of Opensmile feature (Eyben et al., 2010). In Dubagunta et al. (2019), it was shown that voice source-related features, such as linear prediction residual signals, homomorphically filtered voice source signals and zero frequency filtered signals, were better than vocal-tract-related high-frequency features, for depression detection. More recently, articulatory features obtained from acoustic inversion have also been proposed for depression detection (Seneviratne et al., 2020).

### 2.2. Model architectures

Apart from exploring various acoustic features, several studies have also contributed towards improving the backend model architectures. In the past, traditional machine learning methods such as Support Vector Machine (SVM) (Saidi et al., 2020), Gaussian Mixture Models (Sturim et al., 2011) and Random-Forest classifiers (Nasir et al., 2016) have been investigated for depression detection. More recently, deep learning methods for MDD detection have gained popularity due to their superior performance compared to traditional pattern recognition techniques (Shen et al., 2022; Ma et al., 2016; Rejaibi et al., 2022; Chlasta et al., 2019; Harati et al., 2021).

Among the various deep-learning based backend model architectures, Convolutional Neural Networks (CNN) and Recurrent Neural Networks (RNN) such as Gated Recurrent Unit (GRU) and Long Short-Term Memory (LSTM) have been widely applied in depression detection. For example, in Ma et al. (2016), a CNN-LSTM framework called DepAudioNet was proposed, which utilized mel-spectrogram features for depression detection. Another study (Rejaibi et al., 2022) used Mel Frequency Cepstral Coefficients (MFCCs) in combination with a pre-trained RNN model, trained on a Speech Emotion Recognition (SER) task, to achieve improved depression prediction performance. In Harati et al. (2021), the effectiveness of an encoder–decoder structure, where the encoder was pre-trained on Automatic Speech Recognition (ASR) and fine-tuned for depression detection, was investigated. Recently, Shen et al. (2022) proposed an approach that aggregated mel-spectrograms using a NetVLAD network (Arandjelovic et al., 2016) to generate fixed-length segment level embeddings, which were then used to train a GRU model for depression classification. Additionally, in Wang et al. (2022a), an Emphasized Channel Attention, Propagation, and Aggregation in Time-Delay Neural Network (ECAPA-TDNN) model was utilized with MFCC features for depression detection. Furthermore, Wang et al. (2022b) proposed a novel self-supervised learning mechanism called instance-discrimination learning specifically for depression detection.

### 2.3. Speaker-identity and depression detection

Several previous studies have explored the use of speaker-related features for depression detection in the context of speaker identity. Acoustic features such as x-vectors (Ravi et al., 2022a; Egas-López et al., 2022), and other speaker embeddings (Dumpala et al., 2022, 2023) have been found to be effective in diagnosing a speaker’s mental state. However, these features also contain information about the speaker’s identity (Snyder et al., 2018), which can be counterproductive to privacy preservation, a crucial factor in the adoption of digital mental health screening systems (Lustgarten et al., 2020).

### 2.4. Privacy preserving speech processing

While the field of privacy-preserving depression detection is relatively new, there have been some studies that have attempted to address this issue. Notable examples include federated learning (Bn and Abdullah, 2022) and sine-wave speech (Dumpala et al., 2021). However, despite their promise, the application of these methods to low-resource depression detection from speech signals is still in its early stages, and results in significant performance loss (Bn and Abdullah, 2022).

In the past, adversarial speaker normalization has been evaluated in the domain of SER (Yin et al., 2020; Li et al., 2020; Gat et al., 2022). In Yin et al. (2020), the authors perform speaker-invariant domain adaptation on multi-modal features (speech, text, and video). In Li et al. (2020), a gradient reversal technique with an entropy loss is proposed to disentangle emotion and speaker information. In Gat et al. (2022), the authors fine-tune a pre-trained Hubert model (Hsu et al., 2021) with gradient-based adversarial learning. Fine-tuning such models can require large amounts of in-domain data and be computationally intensive. Moreover, these papers utilize IEMOCAP and MSP-Improv datasets which are mono-lingual and consist of acted audio data (Busso et al., 2008, 2016).

In our earlier study (Ravi et al., 2022b), we proposed a method based on adversarial SID loss maximization for depression detection. More recently, Wang et al. (2023) proposed a non-uniform speaker disentanglement method for depression detection based on differential adversarial loss maximization. Although these studies demonstrated a significant improvement in depression detection performance while simultaneously reducing speaker separability, it should be noted that a loss-maximization approach for training neural networks can sometimes be unstable, leading to poor convergence (further explanation in Section 4). Additionally, the privacy attributes of speech representations can be quantified using previously published metrics in the voice-privacy literature (Noé et al., 2020; Tomashenko et al., 2022).

## 3. Privacy preservation and speaker bias in depression detection

In this section, we present the preliminary experiments conducted on the English dataset, DAIC-WOZ (Valstar et al., 2016), to investigate the aspects of privacy preservation and speaker bias in the context of depression detection. The DAIC-WOZ database consists of audio-visual interviews of depressed and non-depressed participants. All experiments in this paper use only the audio portion of the dataset. The database is described in detail in Section 5.

### 3.1. Privacy preservation in depression detection

As mentioned earlier, the use of speaker-identity-related features, such as speaker embeddings, can lead to the identification of individuals. For instance, in our preliminary work, we utilized embeddings from an ECAPA-TDNN model, which is SOTA in SID, to train a naive support vector classifier (SVC) SID system. This setup achieved an SID accuracy of 88% on the DAIC-WOZ dataset(a popular depression detection dataset in English Valstar et al., 2016), even though the ECAPA-TDNN model was originally trained for optimizing depression detection and not speaker prediction (further details in Section 6). This highlights that depression detection frameworks that heavily rely on speaker-identity-related features may compromise the privacy of patients.

### 3.2. Speaker-bias in depression detection

In addition to the well-documented privacy concerns associated with over-reliance on speaker features (Ravi et al., 2022b), another detrimental effect can be overfitting of the model to the speakers in the training set. To investigate this issue, a straightforward approach is to normalize speaker information across all utterances in a dataset by using a voice conversion (VC) system to convert all speakers’ utterances into a single speaker’s voice, and then training the depression classification system on the converted dataset. If there is an improvement in depression classification performance after the single-speaker conversion process compared to the one without VC, it suggests that speaker-identity-related features may introduce bias in depression detection. Therefore, in this section, we conduct a preliminary VC experiment using the DAIC-WOZ dataset.

VQMIVC (Vector Quantization and Mutual Information-Based Unsupervised Speech Representation Disentanglement for One-shot Voice Conversion, Wang et al. (2021)), a SOTA VC system, is used to convert all speakers in the DAIC-WOZ dataset into a single speaker (p334_047). To ensure the quality of the converted utterances, several additional steps are taken. First, each utterance is segmented into non-overlapping 50-s clips, and conversion is applied to each clip, followed by concatenation. Second, to address the issue of audio loudness discrepancy, each segment’s loudness in DAIC-WOZ is scaled to match the maximum loudness of the reference waveform before conversion. In addition, converted audio files were verified manually for quality. Target speaker p334_047 was used because it was provided with the demo of the VC model. Another target speaker was evaluated (p225_038) but the conversion quality was found to be poor.

The DepAudioNet model (Ma et al., 2016) is chosen for major depressive disorder (MDD) classification. Both the baseline and voice conversion (VC) experiments use the same feature processing, model hyperparameters, configurations, and dataset splits, as described in Section 5. The results of the VC experiment are reported in Table 1 in terms of F1-AVG, which is the macro average of the F1-Scores for the two classes — depressed (D) and non-depressed (ND).

Table 1 shows that converting all utterances into a single speaker improves depression classification performance, with the F1-AVG increasing from 0.6081 for the DepAudioNet baseline to 0.6237 for the VC DepAudioNet. This supports the hypothesis that some speaker-related features may introduce bias in depression detection.

The use of voice conversion (VC) to mitigate speaker bias in depression detection may not be a desirable final solution for several reasons. First, even SOTA VC systems can result in loss of content for some speakers (Qian et al., 2022), risking the loss of depression-related information during conversion. Second, dataset-domain discrepancy between the VC training (VCTK Veaux et al., 2016) and the target dataset (e.g., DAIC-WOZ) may still result in preserved speaker information, introducing bias. As mentioned in the VCTK dataset description, VCTK contains accented read speech spoken by native English speakers from the UK, whereas DAIC-WOZ is spontaneous American English speech directed towards a robotic AI assistant. Besides, the two datasets have different channel attributes such as loudness. Therefore, VC systems trained on VCTK but evaluated for DAIC-WOZ may not be 100% successful (Huang et al., 2021). Lastly, converting an entire dataset using VC can be computationally expensive and requires tedious manual verification, making it unfeasible in real-world scenarios.

## 4. Speaker disentanglement for depression detection

**Fig. 1.**
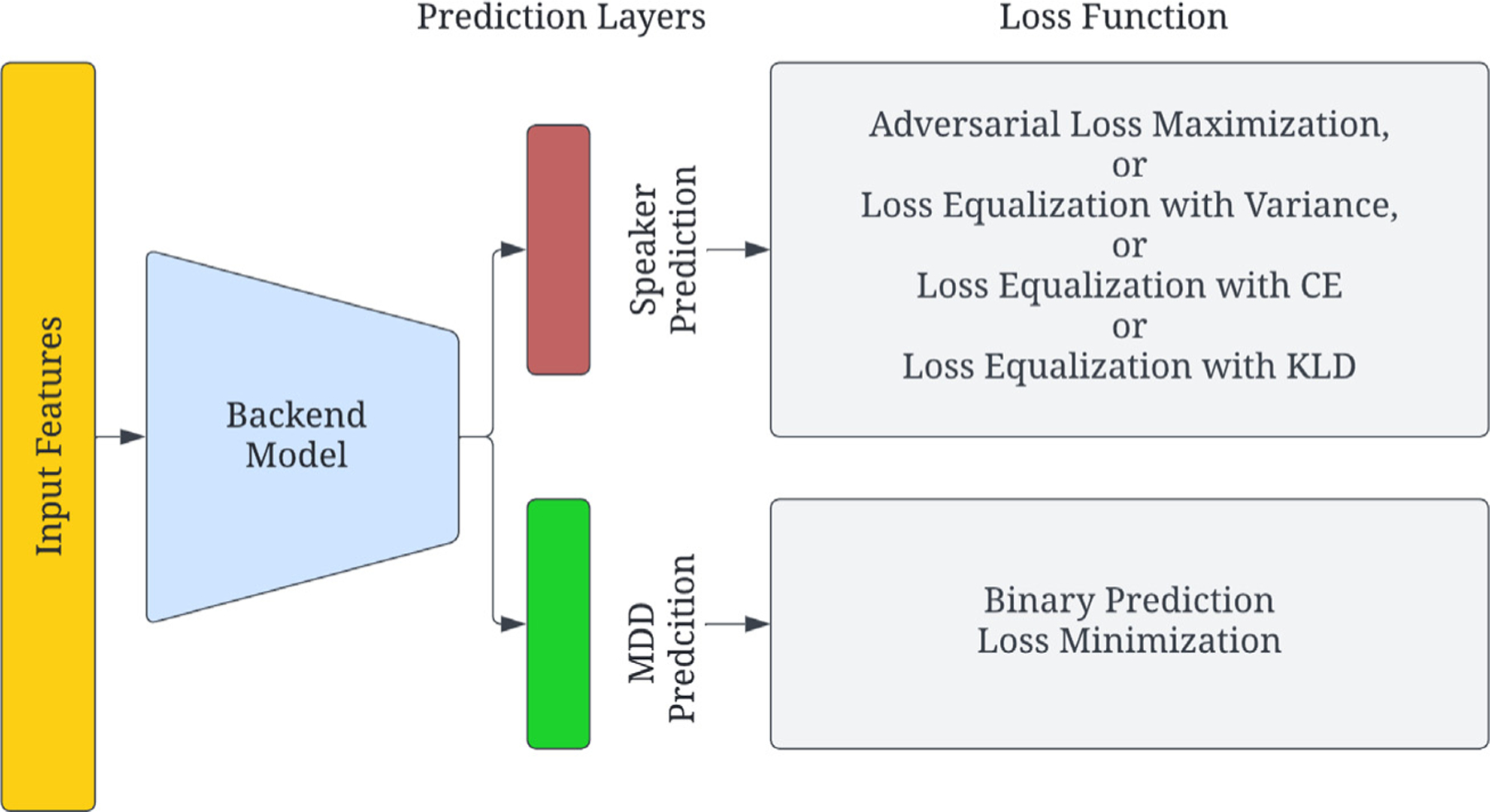
Block diagram representing disentanglement of speaker and depression characteristics. Four methods of speaker disentanglement are proposed.

To mitigate privacy and bias issues as discussed in the previous section, we propose four distinct methods of speaker-disentanglement for depression detection in the form of SID loss manipulation, as shown in Fig. 1.

### 4.1. Adversarial SID-loss maximization

In the first approach, we describe the speaker disentanglement method proposed in our previous work (Ravi et al., 2022b). This method involves an adversarial learning mechanism based on SID loss for speaker-disentangled depression detection, which we refer to as ADV in this paper. Inspired by the domain-adversarial training proposed in Ganin et al. (2016), our approach employs a loss minimization–maximization technique.

Let the number of samples in a training batch be N. The loss used for the prediction of MDD binary labels is:

(1) $$L_{MDD}=-\frac{1}{N}\overset{N}{\underset{n=1}{\sum }}\left[Y_{n}\cdot \text{log}\left(p_{n}\right)+\left(1-Y_{n}\right)\cdot \text{log}\left(1-p_{n}\right)\right]$$

$Y_{n}\in \{0,1\}$ is the class label for the nth sample and $p_{n}$ is the probability that sample n’s label is depressed. If we denote the total number of unique speakers as M, the adversarial loss for speaker ID prediction is defined as -

(2) $$L_{adv}=-\frac{1}{N}\overset{N}{\underset{n=1}{\sum }}\left[\log\frac{\exp\left(x_{n,\hat{n}}\right)}{\sum_{m=1}^{M}\exp\left(x_{n,m}\right)}\right],$$

where $x_{n,m}$ is the score of the nth sample’s speaker ID being predicted as speaker m where m∈1,2,…,M. And, $\overset{ˆ}{n}$ is the coordinate for the ground-truth speaker ID of sample n.

To train the model in an speaker-identity-invariant manner, during optimization, we minimize the depression loss and maximize the speaker prediction loss. This can be written as:

(3) $$L_{total_{-}adv}=L_{MDD}-\lambda \left(L_{adv}\right)$$

where λ is an empirically determined hyperparameter that controls how much of the speaker loss contributes to the total loss. Through this process, we encourage the model to prioritize depression-discriminatory information and reduce its reliance on speaker-specific characteristics, making the model more invariant to changes in speaker-related features.

Although loss maximization has been widely used in speech-related tasks, the adversarial SID loss is unbounded (because of the log-function in Eq. (2)) which can sometimes result in poor model convergence (Xing et al., 2021). In addition, during cross-entropy loss optimization in the SID branch, as shown in Eq. (2), only the probability of the specific speaker $\overset{ˆ}{n}$ corresponding to that sample $x_{n}$ is considered^1^ leaving the other probabilities unused, which can limit the potential of disentangling speaker information.

### 4.2. SID-loss equalization with variance

To overcome the limitations of adversarial loss maximization, a loss equalization-based approach is proposed. Instead of forcing the model to make wrong predictions about speaker identity, equalization methods tend to confuse the model to not be able to distinguish speaker classes through a uniform regularization process similar to an $L_{2}$ norm. The equalization loss is formulated as follows:

(4) $$L_{Evar}=\frac{1}{N}\overset{N}{\underset{n=1}{\sum }}\left[∥\sigma \left(x_{n}\right)-e{∥}^{2}\right]$$

where e=[1/M,1/M,…,1/M] is the vector that assigns equal probability to each speaker in a uniform manner, with length M and $x_{n}$ is the M-dimensional output logit obtained from the model and σ is the softmax function to convert logits to probabilities. Since the new loss term is meant to be minimized, the objective function is defined as follows:

(5) $$L_{\text{total}\_\text{Evar}}=L_{MDD}+\lambda \left(L_{\text{Evar}}\right),$$

In the initial experiments using Eq. (4), it was observed that the model learned to predict the e-vector very easily within a few epochs without learning to disentangle speakers i.e., the speaker prediction branch was overfitting to directly predict the e-vector without tangible speaker disentanglement. We refer to this situation as the “trivial” solution. To avoid this scenario, additive noise (U(0,1)) is injected into the vector e. This method is referred to as Loss equalization with Variance (LEV) in further sections.

### 4.3. SID-loss equalization with cross-entropy

In LEV, loss-equalization is achieved via the $L_{2}$ loss. Alternatively, loss-equalization can also be achieved by minimizing the Cross-Entropy loss between the speaker prediction probabilities and a ones-vector of the same dimension. Mathematically, the equalization loss can be formulated as:

(6) $$L_{Ece}=-\frac{1}{N}\overset{N}{\underset{n=1}{\sum }}\overset{M}{\underset{m=1}{\sum }}\left[y_{n,m}\cdot \text{log}\sigma \left(x_{n,m}\right)+\left(1-y_{n,m}\right)\cdot \text{log}\left(1-\sigma \left(x_{n,m}\right)\right)\right]$$

where $y_{n}=[1,1,\ldots ,1]$ is the M-dimensional target vector and $x_{n}$ is the M-dimensional output logits of the models for the nth sample, respectively. σ is a Softmax function to convert logits to probabilities. Since $y_{n,m}=1$ for all n,m, the above equation can be simplified as -

(7) $$L_{Ece}=-\frac{1}{N}\overset{N}{\underset{n=1}{\sum }}\overset{M}{\underset{m=1}{\sum }}\left[\text{log}\sigma \left(x_{n,m}\right)\right]$$

Therefore, the total loss can be written as -

(8) $$L_{\text{total}\_\text{Evar}}=L_{MDD}+\lambda \left(L_{Ece}\right),$$

This method is referred to as Loss equalization with Cross-Entropy (LECE) in further sections.

### 4.4. SID-loss equalization with KL divergence

Another approach to achieve speaker disentanglement is by manipulating the distribution of the SID-prediction logits. We hypothesize that a uniform distribution for SID logits can help in disentangling speaker identity and MDD characteristics. To achieve this, we propose to minimize the KL-divergence loss between the normalized predicted logits and a uniform vector e. We denote this method as LEKLD in the following sections. The KL-divergence based equalization loss is formulated as:

(9) $$L_{EKL}=L_{KL}\left(x,e\right)=\frac{1}{N}\overset{N}{\underset{n=1}{\sum }}\overset{M}{\underset{m=1}{\sum }}e_{m}\cdot \left(\text{log}\left(e_{m}\right)-\text{log}\left(\sigma \left(x_{n,m}\right)\right)\right)$$

where $x_{n,m}$ and $e_{m}$ stand for the mth element in predicted logits $x_{n}$ and uniform vector e, respectively and σ is the Softmax function. Thus, the final loss with KL-divergence term is computed as:

(10) $$L_{\text{total}\_\text{EKL}}=L_{MDD}+\lambda \left(L_{EKL}\right),$$

## 5. Experimental details

To demonstrate that the proposed framework of speaker disentanglement is invariant to the input features, the backend models, or the datasets, seven acoustic features, three types of model architectures, and two datasets from different languages were investigated. This section provides details about the datasets, input features, feature processing, models, and evaluation metrics employed in this study.

### 5.1. Datasets

Experiments were conducted using two publicly available datasets — the DAIC-WOZ (Valstar et al., 2016), and EATD (Shen et al., 2022). The datasets are described in the following subsections and the datasets’ details are summarized in Table 2.

#### 5.1.1. DAIC-WOZ

The DAIC-WOZ database (Valstar et al., 2016) used in this study comprises audio-visual interviews of 189 participants, both male and female, who underwent psychological distress evaluation. Each participant was assigned a self-assessed depression score using the patient health questionnaire (PHQ-8) method (Kroenke et al., 2009). Only the audio data belonging to the participants were extracted using the provided time labels. The dataset consists of 22.5 h of participant audio data sampled at 16 kHz, with 107 speakers used for training and 35 speakers used for evaluation, following the data partitioning provided in the database description. Models are evaluated using the test data subset and were also evaluated using the validation subset to enable comparison with previous studies (Feng and Chaspari, 2022; Ma et al., 2016; Bailey and Plumbley, 2021).

#### 5.1.2. EATD

The EATD Corpus (Emotional Audio-Textual Depression dataset, Shen et al. (2022)) comprises audio and text transcripts from interviews conducted with 162 Mandarin-speaking participants, including both male and female individuals. Each participant answers three randomly selected questions and completes the SDS questionnaire (Zung, 1965), which is a commonly used screening tool for depression. In this dataset, participants with an SDS score greater than 52 are considered depressed, resulting in a total of 30 depressed volunteers and 132 non-depressed volunteers. For our study, we only utilize the audio portion of the dataset, which has an overall duration of 2.26 h and is sampled at 16 kHz. Data partitioning is done according to the provided database description, with 83 speakers used for training and 79 speakers used for evaluation.

### 5.2. Input features

We explored seven different acoustic features which can be broadly categorized into two groups: low-level and high-level features.

#### 5.2.1. Low-level features

The low-level features employed in this study comprise three commonly used feature sets in previous research on depression detection: (1) Mel-spectrograms, (2) raw audio signals, and (3) OpenSmile features (Eyben et al., 2010). Mel-spectrograms are frame-level features extracted using a Hanning window of length w=1024 samples (64 ms) and a hop size of h=512 samples (32 ms). The dimensionality of Mel-spectrogram features is either 40 or 80, depending on the model size. Raw audio signals are one-dimensional vectors representing the waveform. OpenSmile features consist of 130-dimensional features from the ComparE16 feature set (Schuller et al., 2016), which includes 65 frame-level low-level descriptors and their deltas. All three types of input features are normalized using Mean-Variance normalization for consistency in model training and evaluation.

#### 5.2.2. High-level features

Self-supervised learning (SSL) models have gained popularity in speech-processing tasks due to their ability to leverage large amounts of unlabeled data to learn generic speech patterns that are invariant to downstream tasks. These SSL models can then be fine-tuned or used as feature extractors for specific tasks, such as speech recognition, emotion recognition, etc. In this paper, the SSL models are utilized as feature extractors, and the weights of the pre-trained models are frozen without further fine-tuning. Frame-level representations extracted from the following SSL models are used as input features for depression detection:

- Wav2vec2.0 (Baevski et al., 2020): Features are extracted from the base pre-trained model provided by the fairseq toolkit (Ott et al., 2019), with a hidden dimension of 768. This model was chosen due to its excellent performance in speech-related tasks on the SUPERB benchmark (Yang et al., 2021) and being one of the first SSL models specifically trained for speech processing.
- ContentVec (Qian et al., 2022): ContentVec is an extension of the HuBERT model (Hsu et al., 2021) with speaker disentanglement. ContentVec features capture more content-related information and less speaker-related information, hence the name. Features are extracted using the 100-cluster base model with a hidden dimension of 768, and the extraction process is similar to Wav2vec2.0.
- WavLM (Chen et al., 2022a): WavLM includes a signal reconstruction component and is robust in domain-mismatched scenarios such as noisy conditions. Features are extracted using the base model configuration with a feature dimension of 768.
- Whisper (Radford et al., 2022): Whisper is a recently proposed large-scale, weakly supervised,^2^ pre-trained model for speech recognition that outperforms other SOTA SSL models on speech-recognition tasks. The base English-only model is chosen with a hidden dimension of 512. Extraction is done using the OpenAI toolkit (Brockman et al., 2016).

### 5.3. Models

The paper evaluates three different model architectures for depression detection: CNN-LSTM, ECAPA-TDNN, and LSTM-only. The choice of model architecture is based on the dataset size and/or input feature type. The model architectures are summarized in Table 3. All three models are trained from scratch.

#### 5.3.1. CNN-LSTM

The CNN-LSTM model, inspired by the DepAudioNet framework (Ma et al., 2016), was chosen as one baseline, with implementation based on Bailey and Plumbley (2021). The network parameters, such as the number of hidden layers, learning rate, dropout probability, etc., were chosen empirically. The architecture includes 1D convolutional layers (*Conv1D*) with parameters including channels (C), kernel size (K), and stride (S), and recurrent LSTM layers with a hidden state dimension (H). The number of trainable parameters of CNN-LSTM models is relatively small and pilot experiments with high-level SSL features and CNN-LSTM models resulted in overfitting of the model to the training set despite adopting a smaller learning rate, learning rate decay, and weight decay. Therefore, this architecture was used only with low-level features.

For the DAIC-WOZ dataset, the model with 40-dimensional Mel-spectrograms as input consisted of one *Conv1D* layer (C=128,K=3,S=1) and two unidirectional LSTM layers (H=128). In case of raw-audio signals, two *Conv1D* layers ($C_{1}=128,K_{1}=1024,\;\left.S_{1}=512,C_{2}=128,K_{2}=3,S_{2}=1\right)$ and two LSTM layers (H=128) were used. When 130-dimensional ComparE16 features were used, model comprised of one *Conv1D* layer (C=256,K=3,S=1) and two unidirectional LSTM layers (H=256).

For the EATD dataset and raw-audio signals, two *Conv1D* layers ($C_{1}=128,K_{1}=1024,S_{1}=512,C_{1}=128,K_{2}=3,S_{2}=1$) and two LSTM layers (H=128) were used. With ComparE16 features for the EATD dataset, model comprised of one *Conv1D* layer (C=256,K=3,S=1) and two unidirectional LSTM layers (H=256).

The *Conv1D* layers were followed by ReLU non-linearity, a max-pooling layer with a kernel of size 3 and a dropout layer. For every model configuration, the final prediction layers (fully connected layers, whose inputs were the last-hidden-state of the preceding LSTM layer) generated the predictions for MDD and speaker labels. Based on the number of speakers in the training set, output dimensions for speaker labels were 107 for experiments with DAIC-WOZ and 83 for EATD. For MDD prediction, a sigmoid activation was applied and the binary cross entropy loss was used. For SID branch, cross entropy loss was used without any output activation for ADV, minimum square error loss with softmax activation was used for LEV and point-wise KL divergence loss was used for LEKLD with log-softmax as the activation function.

#### 5.3.2. ECAPA-TDNN

ECAPA-TDNN is a model architecture previously proposed for speaker recognition tasks (Desplanques et al., 2020) and is currently the SOTA in SID. In this paper, to adapt to the smaller training dataset of depression classification and the inherent class-imbalance problems, a modified version of the original ECAPA-model is proposed. Specifically, the kernel (K) and stride (S) of the input convolution layer, the number of channels (C) in the intermediate layers, the attention dimension, the embedding dimension and the dimensions of the prediction layers were empirically modified.

For the DAIC-WOZ dataset and Mel-spectrograms as input, the model consists of one *Conv1D* layer (C=128,K=5,S=1) followed by three SE-Res2Blocks with identical channel dimension, kernel size, and stride as C=128,K=5,S=1. The three SE-Res2Blocks have increasing dilation steps as 2, 3, and 4. In our experiments, it was experimentally found that 80 dimensional Mel-spectrogram performed better compared to 40-dimensional ones. In addition to using Mel-Spectrograms as input features, we investigate the usage of raw-audio signals as input features. In this case, one input convolution layer (C=128,K=1024,S=512) was followed by three SE-Res2Blocks. Dimensions of the SE-Res2Blocks were same as that used with Mel-Spectrograms.

For both Mel-spectrograms and Raw-Audio signal, the attention dimension was 64 and the embedding dimension was 128. The final projection layer is similar to CNN-LSTM architecture but the input to the prediction layers is from the embedding layer (as opposed to the last-hidden-state of the LSTM layer in the CNN-LSTM model).

#### 5.3.3. LSTM-only

The LSTM-only architecture used for high-level features of the DAIC-WOZ dataset in this study consisted of an input LSTM layer with a hidden state dimension of H=256, followed by five hidden LSTM layers with the same hidden state dimension as the input layer. Similar to the CNN-LSTM model, the output of the last-hidden state of the preceding LSTM layer was used as input to the prediction layer. The dimensions of the last prediction layer were dependent on the number of speakers in the training data, as explained earlier in the paper. This architecture was used to process the latent representations obtained from the SSL models, which were used as encoders (feature extractors) in this study.

### 5.4. Evaluation metrics

Every model is evaluated on two aspects — the ability to classify depression status and the ability to protect speaker identity.

#### 5.4.1. Depression detection

Depression detection is evaluated using the macro average F1-score (F1-AVG) of depression (F1-D) and non-depression (F1-ND) classes computed at a speaker level. We opted to report F1-AVG because it provides a balanced representation of both D (Depression) and ND (Non-Depression) prediction capabilities.

#### 5.4.2. Speaker-separability and identification

Inspired by the Voice-privacy literature (Noé et al., 2020; Tomashenko et al., 2022), we use Gain of voice distinctiveness $\left(G_{V\;D}\right)$, measured in dB, and De-Identification Score (DeID), measured in percentage, as metrics to quantify speaker-separability and identification, respectively. A 0 db $G_{V\;D}$ means identical voice distinctiveness before and after disentanglement. A negative $G_{V\;D}$ stands for a decreased speaker distinctiveness and vice versa. In the case of DeID, a score of 100% indicates an optimal de-identification strategy whereas 0% indicates a disentanglement approach that does nothing. Mathematical equations to compute $G_{V\;D}$ and DeID are provided in Appendix.

### 5.5. Training and evaluation scheme

For the DAIC-WOZ dataset, to address data imbalance, the training data were pre-processed using random cropping and sampling techniques, similar to Ma et al. (2016). Each utterance was randomly cropped into fragments of the length of the shortest utterance, and each fragment was further segmented into multiple segments. Segment lengths were set to 3.84 s, which corresponds to 120 frames for Mel-spectrogram, 61 440 samples for raw-audio, 200 frames for Wav2vec2.0 features, and 193 frames for ContentVec, WavLM, and Whisper. A training subset was generated by randomly sampling, without replacement, an equal number of depression and non-depression segments. Five separate models were trained for each experiment using randomly generated training subsets.

In contrast, for the EATD dataset, segments were generated without random cropping and sampling, and the segment length was kept the same as before (3.84 s). Pilot experiments with random cropping and sub-sampling for EATD showed degraded performance, perhaps due to the smaller size of the training dataset compared to DAIC-WOZ. Each experiment was performed by training only one model using all of the training data.

To avoid overfitting the models to the training set the following mechanisms were adopted - (1) random cropping and selection of segments to ensure class imbalance does not influence results, (2) aggregation of 5 models trained with different random seeds to average the effects of random segmentations, (3) reduction of learning rate using a factor of 0.9 when the validation loss does not reduce for two successive epochs and (4) dropout with p=0.6 for LSTM-only, 0.5 for ECAPA-TDNN, and 0.05 for CNN-LSTM models.

At the evaluation stage, segment-level prediction scores are rounded to 0 or 1, representing ‘non-depressed’ or ‘depressed’ classes, respectively. Then, each model generates a speaker-level prediction score by averaging all segment-level scores. For experiments conducted on the DAIC-WOZ dataset where more than one model is trained, 5-model prediction aggregation is performed using two different methods — averaging (5M-AVG) or majority voting (5M-MV). For the averaging method (5M-AVG), speaker-level scores from all models are averaged and rounded for each individual. In contrast, for the majority voting method (5M-MV), speaker-level scores for all models are first rounded, and then a majority vote is taken. All rounding operations use a threshold of 0.5 to determine the final predicted class label for each individual. Moreover, for comprehensive coverage, we include the F1-Score derived from the log-likelihood ratio-based (LLR) aggregation of segment probabilities for the English dataset. For the aggregation of segments to speaker prediction, we utilized an epsilon value of 1e-8.

For the speaker-separability experiments, Probabilistic Linear Discriminant Analysis (PLDA) models are trained using embeddings of 25 speakers (randomly selected). For $G_{V\;D}$ computation, two PLDA models are trained separately — one using embeddings from baseline and the other using embeddings from the disentangled model. On the other hand, for DeID computation, a single PLDA model is trained by combining embeddings from both baseline and disentangled models. Evaluation of $G_{V\;D}$ and DeID is done on the remaining 10 speakers. For each speaker, to reduce computational complexity, 50 segments are randomly chosen using which similarity matrices are generated as per equations described in Appendix A. The Log-likelihood scores in the referenced equations are computed using the trained PLDA models. The experiments are repeated three times using different random seeds and the average $G_{V\;D}$ and DeID are reported.

Lastly, all model hyperparameters, including learning rate, batch size, and learning rate decay, are kept the same for both the baseline and the corresponding disentanglement experiments. The only hyperparameter that varies is the λ parameter, which controls the degree of disentanglement. For baseline experiments, λ is set to 0, while for disentanglement experiments, λ is selected empirically to achieve the desired level of disentanglement in the latent representations.

## 6. Results and discussion

The experimental results are organized and discussed in four stages. First, we present the results for four speaker-disentanglement methods using the DAIC-WOZ validation dataset for all model-feature combinations considered, comparing the baseline methods with no disentanglement with our proposed approach. Following previous studies (Ma et al., 2016; Bailey and Plumbley, 2021; Feng and Chaspari, 2022), our discussion of results is limited to segment-level probability averaging. Second, the performance of the models is presented on the test set of the DAIC-WOZ dataset to show the effectiveness of the method on a held-out test set. Third, we extend the best-performing configuration to the EATD dataset to evaluate the generalizability of our method. Lastly, we compare the best-performing system using our method to the SOTA methods in depression-detection literature.

### 6.1. Speaker disentanglement with DAIC-WOZ

#### 6.1.1. Adversarial loss maximization (ADV)

**Fig. 2.**
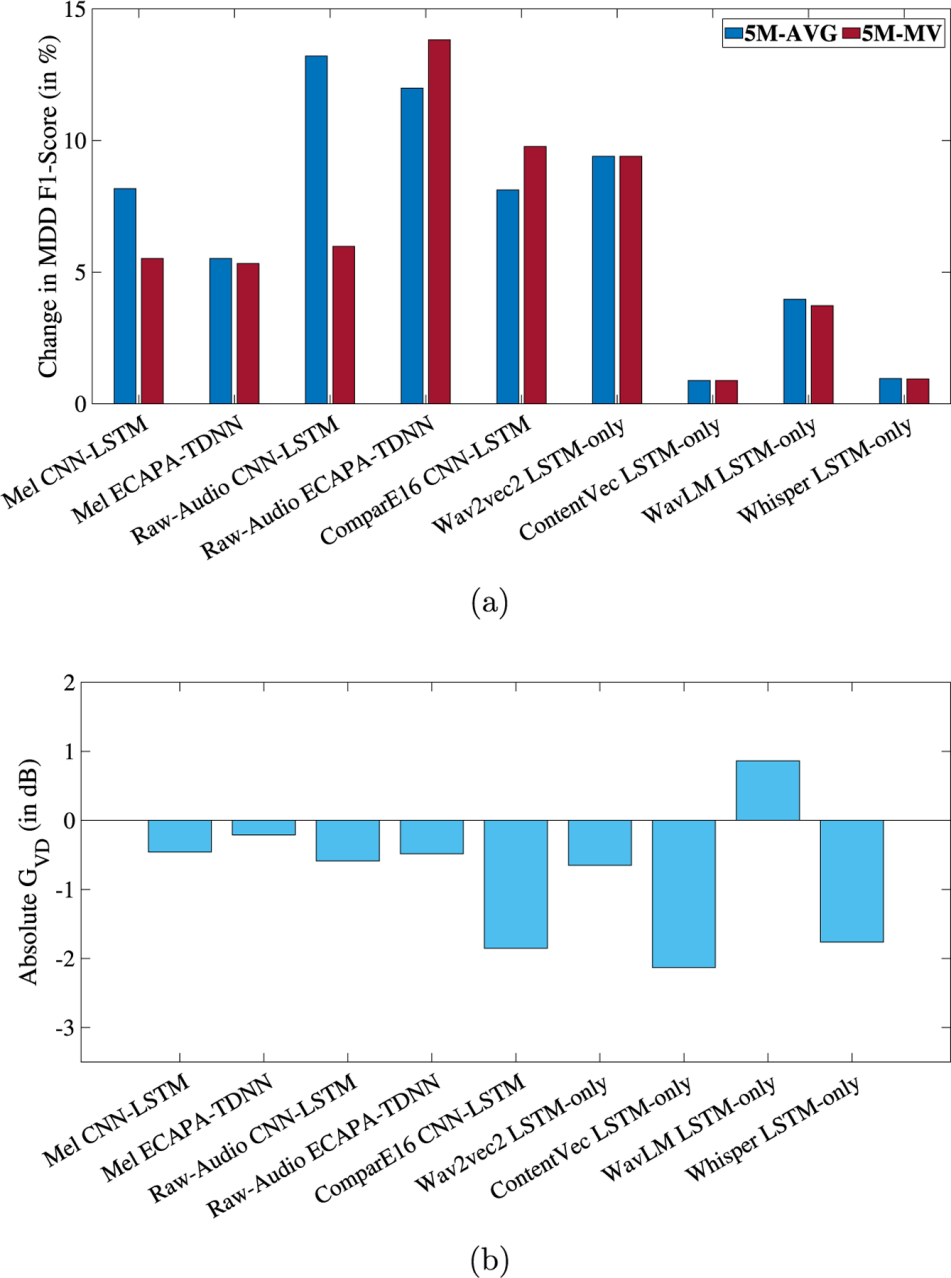
(a) Relative change, in percentage, in MDD classification F1-Score and (b) $G_{V\;D}$ in dB, respectively, for each experiment when speaker disentanglement is applied in the form of ADV. The X-axis of each plot represents the 9 different feature-model combinations. 5M-AVG and 5M-MV refer to the averaging and majority voting aggregation of the 5 models, respectively.

Fig. 2 shows the relative change in MDD classification F1-AVG and the absolute speaker $G_{V\;D}$ (in dB) for each model-feature combination when ADV is applied. Detailed results are presented in the Appendix (Table B.1).

Across all experiments, it was observed that the MDD F1-AVG score increases when speaker disentanglement is applied, while the $G_{V\;D}$ is negative in 8 out of 9 scenarios indicating a reduction in speaker separability. On average, over 9 experiments, there was an improvement of 6.53% in MDD F1-AVG. Improvements in MDD detection were statistically significant (McNemar, 1947) in 6 out of the 9 experiments (relative change obtained with ComparE16, ContentVec, and Whisper were not statistically significant). Although positive trends were observed in all experiments, results for Raw-Audio with ECAPA-TDNN, ContentVec with LSTM-only, and WavLM with LSTM-only are selectively discussed below.

In the case of the ECAPA-TDNN model is trained with raw-audio signals, the baseline setup without disentanglement achieves an F1-AVG score of 0.6196 (5M-AVG) and 0.6941 (5M-MV). Recall that 5M-AVG and 5M-MV refer to the averaging and majority voting aggregation of the 5 models, respectively, as described in Section 5. The best-performing configuration is obtained when adversarial loss maximization is applied to the ECAPA-TDNN model with raw audio signals as input. The F1-AVG increases to 0.6939 (5M-AVG) and 0.7900 (5M-MV). This configuration has a $G_{V\;D}$ of −0.48 dB which indicates a reduction in speaker separability when ADV is applied and a DeID of 22% that indicates a partially successful masking of speaker identities.

ContentVec with LSTM-only, on the other hand, resulted in smaller improvements when speaker disentanglement was applied. For example, the improvement in F1-AVG is only 0.88% for both 5M-AVG and 5M-MV. Although the improvements in F1-AVG were small, the $G_{V\;D}$ was −2.13 dB, the lowest among all features with a DeID of 42.5%. It is possible that because ContentVec already includes 3 speaker disentanglement stages, features extracted from it have lost much speaker-identity-related information, and therefore, another disentanglement approach improves depression detection performance only marginally but can severely degrade speaker separability.

In contrast, it was observed that Speaker $G_{V\;D}$ was negative for all scenarios except WavLM LSTM-only experiments where GvD was 0.863 dB. However, the DeID for WavLM was 83.55% indicating that although the speaker identities were successfully masked when ADV was applied (because of positive DeID), they were still separable (positive $G_{V\;D}$).

#### 6.1.2. Loss Equalization with Variance (LEV)

**Fig. 3.**
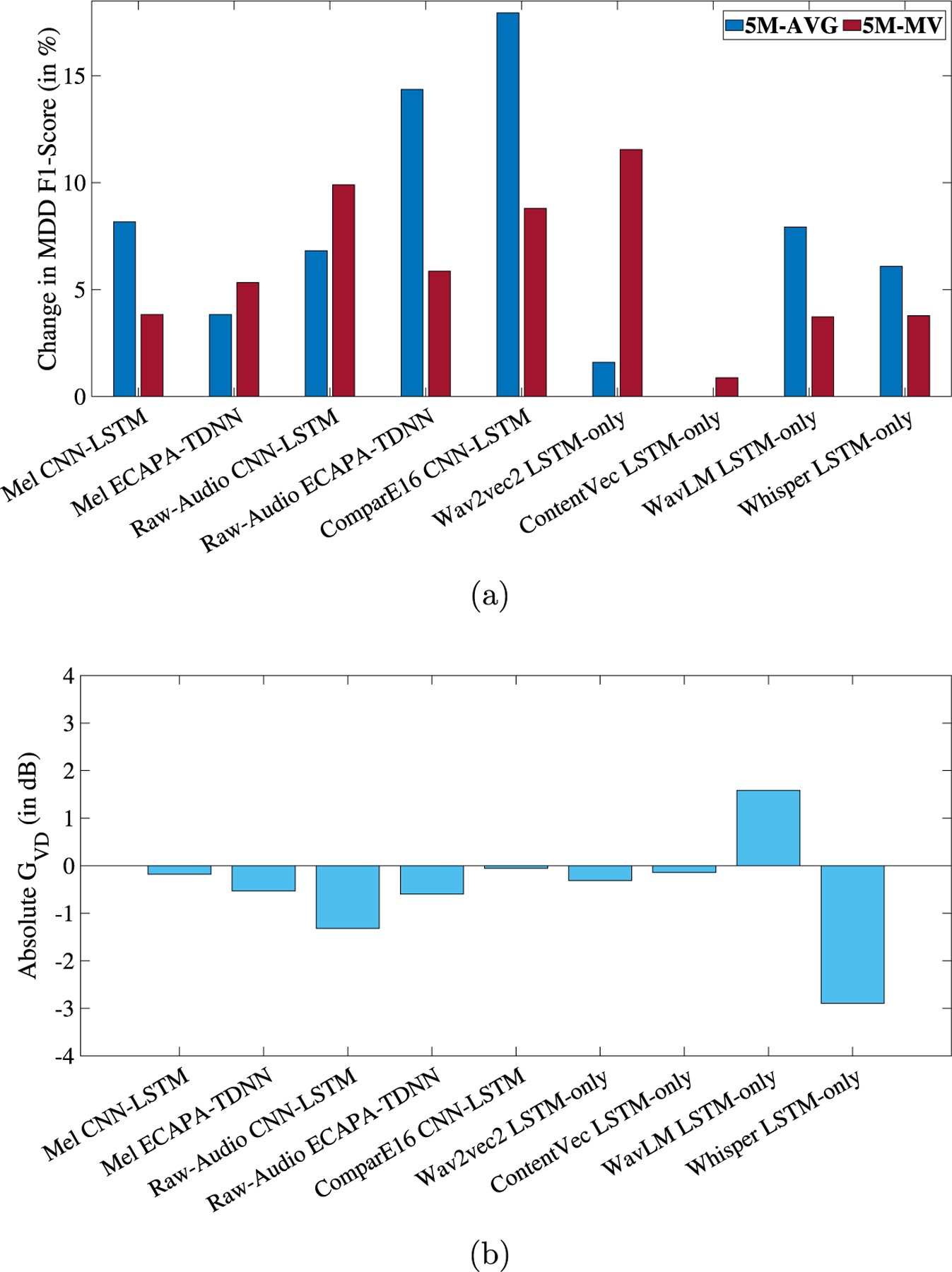
(a) Relative change, in percentage, in MDD classification F1-Score and (b) $G_{V\;D}$ in dB, respectively, for each experiment when speaker disentanglement is applied in the form of LEV. The X-axes represent the 9 different feature-model combinations. 5M-AVG and 5M-MV refer to the averaging and majority voting aggregation of the 5 models, respectively.

Fig. 3 shows the relative change in MDD classification F1-AVG and the absolute speaker $G_{V\;D}$ (in dB) for LEV. Detailed results are presented in the Appendix (Table B.2). Similar to ADV, this loss function results in improvements in MDD detection across all 9 experiments, with an average improvement in F1-AVG of 6.69%. This is accompanied by a negative $G_{V\;D}$ in 8 out of the 9 experiments. Improvements in MDD detection were statistically significant (McNemar, 1947) in 5 out of the 9 experiments (relative change obtained with Raw-Audio, ComparE16, ContentVec, and Whisper were not statistically significant). For LEV, we discuss results from Wav2vec2 LSTM-only, ComparE16 CNN-LSTM, Whisper LSTM-only, and WavLM LSTM-only.

In the case of LEV, Wav2vec2 features with LEV result in the best MDD classification performance. For the baseline model without disentanglement, the F1-AVG scores are 0.6830 (5M-AVG) and 0.6830 (5M-MV). When the proposed method with a hyperparameter value of λ=5e-3 is applied, the F1-AVG increases to 0.6939 (5M-AVG) and 0.7619 (5M-MV). For this case, the $G_{V\;D}$ is −0.3126 dB, and the DeID is 30.41%. A negative $G_{V\;D}$ further shows that the disentangled speaker representations are less separable than their baseline counterparts.

The highest improvements in MDD detection are observed when ComparE16 features are used, with a 17.94% increase in F1-AVG (5M-AVG), from 0.5791 for the baseline to 0.683 for the proposed method (λ=2e-4). The $G_{V\;D}$ for this model is −0.0551 dB whereas the DeID is 79.62% indicating a successful speaker-identity masking mechanism but only a small reduction in speaker-separability.

The lowest $G_{V\;D}$ of −2.589 dB is achieved in LEV when Whisper-base features are used with the LSTM-only model. This feature also has a high DeID of 84.49%. Lastly, similar to ADV, WavLM with LSTM-only and LEV results in the highest $G_{V\;D}$ of 1.5854 dB but has a DeID of 72.71%. Same as before, although this points to a (partially) successful speaker-identity masking method, the disentangled speaker representations are more separable than the baseline embeddings.

#### 6.1.3. Loss Equalization with Cross-Entropy (LECE)

**Fig. 4.**
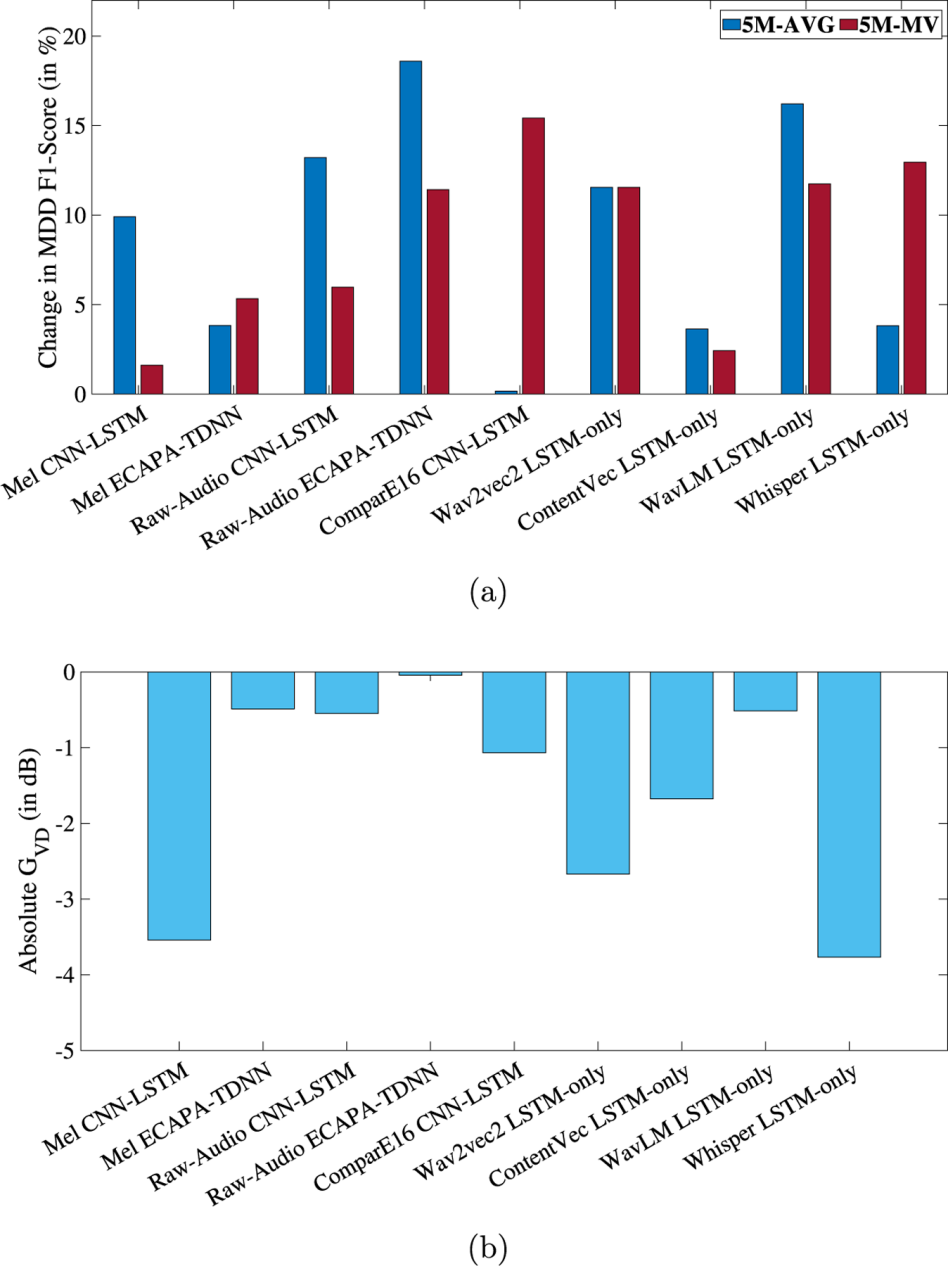
(a) Relative change, in percentage, in MDD classification F1-Score and (b) $G_{V\;D}$ in dB, respectively, for each experiment when speaker disentanglement is applied in the form of LECE. The X-axes represent the 9 different feature-model combinations. 5M-AVG and 5M-MV refer to the averaging and majority voting aggregation of the 5 models, respectively.

Fig. 4 shows the relative change in MDD classification F1-AVG and the absolute speaker $G_{V\;D}$ (in dB) for LECE. Detailed results are presented in the Appendix (Table B.3). Similar to ADV and LEV, this loss function results in improvements in MDD detection across all 9 experiments, with an average improvement in F1-AVG of 8.86%. In contrast to before, a negative $G_{V\;D}$ is observed in all 9 experiments. Improvements in MDD detection were statistically significant (McNemar, 1947) in 4 out of the 9 experiments (relative change obtained with Raw-Audio, ContentVec, WavLM, and Whisper were not statistically significant). For LECE, we discuss results from ComparE16 CNN-LSTM, Raw-Audio ECAPA-TDNN, and Whisper LSTM-only.

ComparE16 features when used with CNN-LSTM features achieved the best MDD classification performance. In the baseline model without disentanglement, the F1-AVG scores are 0.5791(5M-AVG) and 0.6941 (5M-MV). When the proposed method with a hyperparameter value of λ=1e-7 is applied, the F1-AVG increases to 0.5800 (5M-AVG) and 0.8011 (5M-MV). For this case, the $G_{V\;D}$ is −1.0688 dB, and the DeID is 85.10%. A negative $G_{V\;D}$ along with a high DeID shows that identity has been successfully masked and that the disentangled speaker representations are less separable than the corresponding baseline representations.

The highest improvement in F1-Score is observed when Raw-Audio signals are used to train the ECAPA-TDNN model. The baseline F1-AVG score improves by 18.60%, from 0.6196 (5M-AVG) to 0.7348. Although negative, this feature-model combination has the highest $G_{V\;D}$ of −0.0446 dB with a corresponding DeID of 15.62%.

Similar to LEV, Whisper-base features with the LSTM model resulted in the lowest $G_{V\;D}$ of −3.767 dB and a DeID of 86.09%.

#### 6.1.4. Loss Equalization with KLD (LEKLD)

**Fig. 5.**
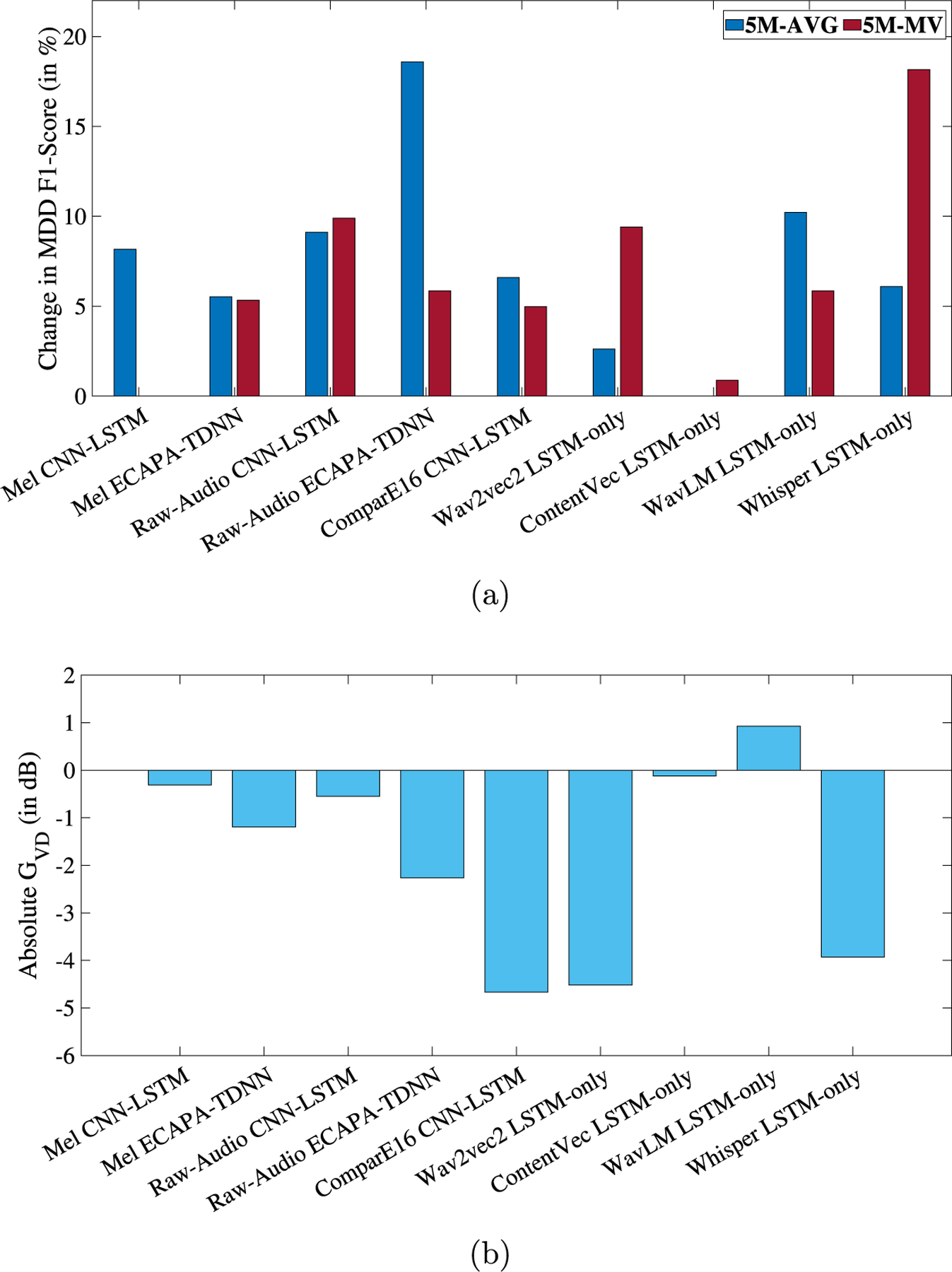
(a) Relative change, in percentage, in MDD classification F1-Score and (b) $G_{V\;D}$ in dB, respectively, for each experiment when speaker disentanglement is applied in the form of LEKLD. The X-axes represent the 9 different feature-model combinations. 5M-AVG and 5M-MV refer to the averaging and majority voting aggregation of the 5 models, respectively.

Fig. 5 shows the relative change in MDD and the absolute speaker $G_{V\;D}$ (in dB) for LEKLD with detailed results in the Appendix (Table B.4). As seen before in ADV, LEV, and LECE, every experiment leads to an improvement in MDD detection performance with an average improvement in MDD F1-AVG by 7.07% and a negative $G_{V\;D}$ is 8 out of 9 experiments. Improvements in MDD detection were statistically significant (McNemar, 1947) in 7 out of the 9 experiments (relative change obtained with ComparE16 and ContentVec were not statistically significant). In this method, we discuss the results from Whisper LSTM-only, Raw-Audio ECAPA-TDNN, WavLM LSTM-only, and ComparE16 CNN-LSTM.

In the case of LEKLD, the best-performing model is the Whisper LSTM-only model with speaker disentanglement. The baseline F1-AVG of 0.6438 (5M-AVG), 0.6686 (5M-MV) increases by 6.09% and 18.16% to 0.6830 (5M-AVG), 0.7900 (5M-MV), respectively when the proposed method is applied (λ=1e-5). For this model-feature combination, the $G_{V\;D}$ is −3.93 dB and the corresponding DeID is 69.42%.

Further, the ECAPA-TDNN model trained with Raw-Audio signals achieves the highest improvement in MDD detection with an improvement of 18.59% in F1-AVG (5M-AVG) from 0.6196 for the baseline to 0.7348 for the proposed method (λ=5e-3). The $G_{V\;D}$ for this model is −2.26 dB and the DeID is 29.56%.

Similar to ADV and LEV, the $G_{V\;D}$ for WavLM was positive (0.9268 dB). However, the DeID for the same feature was 75%. Again, this shows that LEKLD in this scenario can successfully mask speaker-identity but the disentangled representations are more separable than before. In contrast, ComparE16 features with the CNN-LSTM model achieved the lowest $G_{V\;D}$ of −4.66 dB with a DeID of 62.68%.

#### 6.1.5. Results on held-out test set

To evaluate the effectiveness of the proposed methods on a held-out test set, we test the best-performing models using the test set of the DAIC-WOZ dataset. The results in terms of average macro-F1-Score are presented in Table 4.

The proposed speaker disentanglement method improved performance for all systems. The highest overall performance of 0.5529 was obtained using the ComparE16 features and CNN-LSTM model when LECE was applied and the highest improvement of 18% in performance was obtained with Whisper LSTM-only when LEKLD was applied (F1-Score improved from 0.4323 to 0.5116). Improvements in MDD detection were statistically significant (McNemar, 1947) for ComparE16 features with CNN-LSTM but not for Whisper features with the LSTM-only model.

#### 6.1.6. Summary of DAIC-WOZ results

The results of our experiments, which include 9 experiments involving seven different input features and three model architectures, consistently demonstrate that speaker disentanglement improves depression detection performance while simultaneously degrading speaker identification and separability (improvements are statistically significant in 22 out of the 36 experiments). Among the proposed methods, ComparE16 features with CNN-LSTM achieved the highest F1-AVG for MDD detection at 80% when LECE was applied. ADV with Raw-Audio/ECAPA-TDNN and LEKLD with Whisper/LSTM-only achieved the second-best F1-AVG of 79%. The consistent outcome in our experiments, when ContentVec features were used for speaker disentanglement, indicated that this approach consistently yielded the smallest improvements in MDD depression detection. This suggests that when applying speaker disentanglement, features that have already lost a significant amount of speaker-related information tend to result in smaller enhancements.

In terms of privacy attribute DeID, the score was the lowest for Mel-Spectrogram features (DeID = 1.9%) when used with ECAPA-TDNN showing the robustness of ECAPA-TDNN models in extracting speaker-related information from Mel-Spectrograms. In contrast, Whisper/LSTM-only with ADV achieved the highest DeID scores of 90.29% suggesting that large-scale models pre-trained to optimize speech-recognition performance may contain some speaker information that is irrelevant for downstream tasks which can easily be disentangled.

Regarding $G_{V\;D}$, the utilization of ComparE16 features in conjunction with the CNN-LSTM model, along with the application of LEKLD for speaker disentanglement, yielded the lowest $G_{V\;D}$ score, which was recorded at −4.66 dB. This outcome suggests that the proposed framework can effectively diminish the capability of prosodic features to distinguish between speakers. Conversely, when employing WavLM features for speaker disentanglement via LEV, we observed the highest value of 1.59 dB, despite the presence of a high DeID score (72.71%). This suggests that, although our proposed method obscured speaker identities, WavLM features demonstrated considerable resilience, allowing the resulting embeddings to remain distinguishable.

### 6.2. EATD- Speaker disentanglement

To evaluate the generalizability of the proposed speaker-disentanglement method to a different language, we applied it to the EATD dataset. We conducted experiments using the ADV method with CNN-LSTM/Raw-Audio features the LECE method with ComparE16 features and the CNN-LSTM model. Using larger models such as the ECAPA-TDNN or SSL features such as Whisper-multilingual (Radford et al., 2022) as baselines resulted in poor performance due to issues such as model overfitting (a small dataset size) or domain mismatch (multilingual to Mandarin). The results of these experiments are presented in Table 5.

From the table, we can see that when ADV was applied to the CNN-LSTM model trained on Raw-audio, the F1-AVG for MDD prediction increased by 11.99%, from 0.6430 for the baseline model to 0.7201 for the proposed method (λ=3e-5). In contrast, for the LECE method and ComparE16/CNN-LSTM, the performance increased by 2.86% from 65.23% for the baseline model to 0.6710% (λ=4e-3). Improvements in MDD detection were statistically significant (McNemar, 1947) for the ComparE16-CNN-LSTM model but not for the Raw-Audio-CNN-LSTM model. Similar to the DAIC-WOZ dataset, an increase in MDD prediction performance is accompanied by a negative $G_{V\;D}$ and positive DeID. For Raw-Audio/CNN-LSTM, the $G_{V\;D}$ is −0.88 dB with a corresponding DeID of 51.21% and for ComparE16/CNN-LSTM, the $G_{V\;D}$ is −0.1635 dB with a DeID of 8.71%.

In contrast to the DAIC-WOZ dataset, ADV performs better than LECE both on MDD classification and speaker identity preservation. However, overall, these results indicate that speaker-identity-related information is a challenging problem in multiple datasets and our proposed methods have the potential to mitigate these challenges effectively.

### 6.3. SOTA comparison

The SOTA results for depression detection in terms of F1-Score are presented in Table 6.

For the DAIC-WOZ dataset, the proposed method (LECE with CNN-LSTM-only/ComparE16) results in an F1-AVG of 0.80. The method outperforms the best audio-only models in the literature, the Vowel-based method, by 14.28%, and NUSD, by 8.85%. Similarly, for the EATD datasets, the CNN-LSTM model trained with raw-audio signals and ADV results in an F1-AVG of 0.7201, which outperforms an audio-only BiGRU model by 9.1% and also outperforms methods that combine text and audio features (Shen et al., 2022).^3^

## 7. Summary and conclusion

In previous studies, features such as x-vectors and other speaker embeddings have been shown to be effective for depression detection. However, these features also contain speaker-identity information, which can compromise the privacy of an MDD diagnosis system, an important consideration for the adoption of speech-based assessment methods. Consequently, this raises the question of whether depression detection can be achieved in a speaker-invariant manner, without relying heavily on speaker-identity features.

In this paper, we propose a framework for disentangling speaker identity and depression status in order to achieve speaker-identity invariant models for depression detection. Our proposed methods demonstrate improved MDD classification performance across multiple features, models, and two datasets (English and Mandarin). In comparison to SOTA methods from the literature, our methods outperformed them on both datasets. These results indicate that when attributes of a speaker’s identity that are irrelevant to a subject’s mental state are partially normalized, depression diagnosis is more accurate while also enhancing privacy.

Although the proposed method demonstrates strong results, there are some limitations. Firstly, the sensitivity of the method to hyperparameters and the time-consuming nature of hyperparameter tuning may pose challenges in practical implementation. Secondly, the effectiveness of the proposed methods on larger datasets with greater participant diversity needs to be further investigated to ensure generalizability. Lastly, in some cases, while individual model performance was not significantly impacted by the proposed methods, their combination with model aggregations (averaging and majority voting) yielded better results. A more in-depth analysis of such model behavior is warranted in future research.

Additionally, it would be valuable to investigate the specific aspects of speaker-related information that are relevant or irrelevant to depression detection. This could provide insights into the optimal representation of speech features for depression detection while considering the trade-off between privacy and diagnostic accuracy.

## Figures and Tables

**Table 1:** Depression detection performance in terms of F1-AVG on the DAIC-WOZ dataset, with and without voice conversion (VC), using the DepAudioNet model trained using Mel-Spectrograms.

Experiment	F1-AVG
DepAudioNet (Ma et al., 2016)	0.6081
DepAudioNet+VC	0.6237

**Table 2:** Summary of datasets used in this paper. Cases refers to ‘depressed’ class and Controls is ‘non-depressed’ class.

	DAIC-WOZ	EATD
Language	English	Mandarin
Number of participants	142	162
Gender	M&F	M&F
Cases/Controls	42/100	30/132
Sampling rate (Hz)	16000	16000
Total duration (Hours)	22.56	2.26

**Table 3:** Summary of Model architectures used in this paper. ‘*Conv*’ indicates convolutional layer. ‘*LSTM*’ indicates Long Short-term Memory Layer. ‘*FC*’ indicates fully connected layer. The number of layers and dimensions of each varies with the dataset size and/or input features.

Model architecture	Initial layers	Hidden layers	Output layer
CNN-LSTM	*Conv*	*LSTM*	*FC*
ECAPA-TDNN	*Conv*	Time-Dilated *Conv*	*FC*
LSTM-only	*LSTM*	*LSTM*	*FC*

**Table 4:** Results, in terms of F1-Score, for speaker disentanglement through ADV, LEV, LECE, and LEKLD using the test set of DAIC-WOZ dataset. The best F1-Score is bold-faced for each experiment.

Model	Disentanglement method				
	No	ADV	LEV	LECE	LEKLD
Mel Spectrogram CNN-LSTM	0.4101	0.4346	**0.4623**	0.4402	0.4428
Mel Spectrogram ECAPA-TDNN	0.4530	0.4670	0.4751	0.4698	**0.4894**
Raw-Audio CNN-LSTM	0.5107	0.4987	0.4990	**0.5253**	0.4982
Raw-Audio ECAPA-TDNN	0.4264	0.4685	**0.4710**	0.4489	0.4461
Compare16 CNN-LSTM	0.5152	0.4609	**0.5153**	0.4603	0.4983
Wav2vec2 LSTM-Only	0.4926	0.5440	0.5401	0.5223	**0.5440**
ContentVec LSTM-Only	0.4986	0.5129	0.5151	**0.5529**	0.5317
WavLM LSTM-Only	0.4412	**0.5351**	0.5191	0.5075	0.5202
Whisper LSTM-Only	0.4323	0.5027	**0.5116**	0.4672	0.4642

**Table 5:** Results, in terms of F1-AVG, Confusion-Matrix, $G_{V\;D}$ and DeID, speaker disentanglement through ADV and LECE using the development set of EATD dataset. TN, FP, FN, and TP are True Negative, False Positive, False Negative, and True Positive, respectively. The best F1-Score is bold-faced.

Feature-Model	Speaker disentanglement	# Params	F1-AVG	Confusion matrix				*G*_*V D*_ (in dB)	DelD (in %)
				TN FP		FN	TP		
Raw-Audio	No	445k	0.643	62	6	7	4	-	-
CNN-LSTM	ADV (*α* = 3*e*-5)	456k	**0.720**	62	6	5	6	−0.8827	51.21
ComparE16	No	1.15M	0.652	66	2	8	3	-	-
CNN-LSTM	LECE (*α* = 4*e*-4)	1.18M	0.671	64	4	7	4	−0.1635	8.71

**Table 6:** Comparison in terms of F1-Scores of SOTA MDD-prediction methods from literature for DAIC-WOZ and EATD datasets and our proposed method. Best result is bold-faced.

Dataset	Method	MDD F1
DAIC-WOZ	DepAudioNet (Ma et al., 2016)	0.6081
	FVTC-CNN (Huang et al., 2020)	0.6400
	CNN-LSTM (Dumpala et al., 2022)	0.6850
	SpeechFormer (Chen et al., 2022b)	0.6940
	Vowel-based (Feng and Chaspari, 2022)	0.7000
	NUSD (Wang et al., 2023)	0.7349
	Proposed	**0.8000**
EATD	BiGRU+Text (Shen et al., 2022)	0.6500
	BiGRU+Audio (Shen et al., 2022)	0.6600
	RoBERTa+BiLSTM+Text (Zhang et al., 2022)	0.6900
	BiGRU, Fusion Speech + Text (Shen et al., 2022)	0.7100
	Proposed	**0.7201**

## Data Availability

Data used in this study is publicly available and code/models will released upon request.

## Footnotes

1 Numerator contains only the probability of the target speaker, while the denominator uses probabilities for all coordinates. Because the denominator acts as a normalizer, its value is shared across all coordinates. Consequently, the numerator becomes the primary contributor to the loss term for the given target speaker.

2 Whisper pretaining is not self-supervised but has been included in this section for comparison with other large-scale pre-trained speech models.

3 Previous studies using the EATD dataset report results using only F1-D instead of F1-AVG. Experiments to obtain F1-AVG results from publicly available code repositories resulted in an inferior F1-AVG of ~0.4.

## Appendix A. $G_{VD}$ And De-ID calculation

The following equations define $G_{V\;D}$ and DeID as described in (Noé et al., 2020; Tomashenko et al., 2022).

(A.1)
$$G_{VD}=10{\text{log}}_{10}\frac{D_{diag}\left(M_{dd}\right)}{D_{diag}\left(M_{oo}\right)}$$

(A.2)
$$\text{DeID}=1-\frac{D_{diag}\left(M_{od}\right)}{D_{\text{diag }}\left(M_{oo}\right)}$$

where $M_{dd},M_{oo}$ and $M_{od}$ are voice similarity matrices and $D_{diag}(M)$ is called the diagonal dominance. In this paper, o stands for the baseline (original) model and d stands for the disentangled model. A voice similarity matrix $M_{AB}=(M(i,j){)}_{1\leq i\leq N,1\leq j\leq N}$ is defined for an N speakers set where each entry M(i,j) defines the similarity between speaker i and j, calculated as:

(A.3)
$$M_{AB}\left(i,j\right)=sigmoid\left(\frac{1}{n_{i}n_{j}}\underset{\begin{array}{c} 1\leq k\leq n_{i}\;and\;1\leq l\leq n_{j}\\ k\neq l\text{ if }i=j \end{array}}{\sum }\text{LLR}\left(x_{k}^{\left(i\right)},x_{l}^{\left(j\right)}\right)\right)$$

where $LLR\left(x_{k}^{\left(i\right)},x_{l}^{\left(j\right)}\right)$ is the log-likelihood-ratio obtained from Probabilistic Linear Discriminant Analysis (PLDA) model between segment k from speaker i and segment l from speaker $j.n_{i}$ and $n_{j}$ are number of segments from speaker i and speaker j, respectively. A and B denoted the models from which speaker representations $x_{k}^{\left(i\right)}$ and $x_{l}^{\left(j\right)}$ are taken, respectively.

The diagonal dominance is defined as the absolute difference between average diagonal and off-diagonal elements as follows:

(A.4)
$$D_{\text{diag}}\left(M\right)=\left|\underset{1\leq i\leq N}{\sum }\frac{M\left(i,i\right)}{N}-\underset{\underset{j\neq k}{1\leq j\leq N\;and\;1\leq k\leq }}{\sum }\frac{M\left(j,k\right)}{N\left(N-1\right)}\right|$$

## Appendix B. Speaker disentanglement in DAIC-WOZ

Detailed results of three speaker disentanglement methods using the DAIC-WOZ validation dataset are presented in this section.

**Table B. 1:** Results, in terms of F1-Score, Confusion-Matrix, $G_{V\;D}$ and DeID, for speaker disentanglement through ADV using the development set of the DAIC-WOZ dataset. The highlighted row (Δ) for each feature-model configuration indicates the relative change in performance of that model without disentanglement versus our proposed method. The best F1-Score is bold-faced.

Input feature (Seq.len × Num. of features)	Model architecture	Speaker disentanglement	Model parameters	5-Models logit average							5-Models majority voting							F1-AVG (LLR)	*G_V D_* (in dB)	DeID in (%)
				F1-Score			Confusion Matrix				F1-Score			Confusion Matrix						
				F1(Avg)	F1(ND)	F1(D)	TN	FP	FN	TP	F1(Avg)	F1(ND)	F1(D)	TN	FP	FN	TP			
Mel-Spectrogram (120 × 40), (120 × 80)	CNN-LSTM	No	280k	0.6081	0.6977	0.5185	15	8	5	7	0.6578	0.7556	0.5600	17	6	5	7	0.6173	-	-
		Yes (*α* = 4e-5)	293k	0.6578	0.7556	0.5600	17	6	5	7	0.6941	0.7727	0.6154	17	6	4	8	0.6274	−0.4584	14.01
	*Δ* (in %)	-	-	8.17	8.30	8.00	-	-	-	-	5.52	2.26	9.89	-	-	-	-	1.64	-	-
	ECAPA-TDNN	No	515k	0.6578	0.7556	0.5600	17	6	5	7	0.7086	0.8085	0.6087	19	4	5	7	0.5425	-	-
		Yes (*α* = 5e-6)	529k	0.6941	0.7727	0.6154	17	6	4	8	0.7464	0.8261	0.6667	19	4	4	8	0.5425	−0.2118	3.69
	*Δ* (in %)	-	-	5.52	2.26	9.89	-	-	-	-	5.33	2.18	9.53	-	-	-	-	0.00	-	-
Raw-Audio (61 440 × 1)	CNN-LSTM	No	445k	0.6259	0.7755	0.4762	19	4	7	5	0.6686	0.7917	0.5455	19	4	6	6	0.6182		
		Yes (*α* = 3e-6)	459k	0.7086	0.8085	0.6087	19	4	5	7	0.7086	0.8085	0.6087	19	4	5	7	0.6429	−0.5868	55.83
	*Δ* (in %)	-	-	13.21	4.26	27.82	-	-	-	-	5.98	2.12	11.59	-	-	-	-	4.00	-	-
	ECAPA-TDNN	No	595k	0.6196	0.7391	0.5000	17	6	6	6	0.6941	0.7727	0.6154	17	6	4	8	0.5949	-	-
		Yes (*α* = 2e-4)	609k	0.6939	0.8163	0.5714	20	3	6	6	**0.7900**	0.8800	0.7000	22	1	5	7	0.5585	−0.4843	22.32
	*Δ* (in %)	-	-	11.99	10.45	14.28	-	-	-	-	13.82	13.89	13.75	-	-	-	-	−6.12	-	-
ComparE16 (384 × 130)	CNN-LSTM	No	1.15M	0.5791	0.7234	0.4348	17	6	7	5	0.6941	0.7727	0.6154	17	6	4	8	0.4804	-	-
		Yes (*α* = 5e-3)	1.18M	0.6261	0.8077	0.4444	21	2	8	4	0.7619	0.8571	0.6667	21	2	5	7	0.4643	−1.8526	68.37
	*Δ* (in %)	-	-	8.12	11.65	2.21	-	-	-	-	9.77	10.92	8.34	-	-	-	-	−3.35	-	-
Wav2Vec2.0-base (200 × 768)	LSTM-only	No	3.6M	0.6830	0.7826	0.5833	18	5	5	7	0.6830	0.7826	0.5833	18	5	5	7	0.5333	-	-
		Yes (*α* = 4e-6)	3.7M	0.7472	0.8627	0.6316	22	1	6	6	0.7472	0.8627	0.6316	22	1	6	6	0.5333	−0.6503	52.43
	*Δ* (in %)	-	-	9.40	10.24	8.28	-	-	-	-	9.40	10.24	8.28	-	-	-	-	0.00	-	-
Contentvec-100 (193 × 768)	LSTM-only	No	3.6M	0.7287	0.7907	0.6667	17	6	3	9	0.7287	0.7907	0.6667	17	6	3	9	0.4804	-	-
		Yes (*α* = 1e-2)	3.7M	0.7351	0.7805	0.6897	16	7	2	10	0.7351	0.7805	0.6897	16	7	2	10	0.3966	−2.1326	42.50
	*Δ* (in %)	-	-	0.88	−1.29	3.45	-	-	-	-	0.88	−1.29	3.45	-	-	-	-	−17.44	-	-
WavLM-base (193 × 768)	LSTM-only	No	3.6M	0.6429	0.7143	0.5714	15	8	4	8	0.6941	0.7727	0.6154	17	6	4	8	0.5333	-	-
		Yes (*α* = 4e-7)	3.7M	0.6684	0.7442	0.5926	16	7	4	8	0.7200	0.8000	0.6400	18	5	4	8	0.6749	0.863	83.55
	*Δ* (in %)	-	-	3.97	4.19	3.71	-	-	-	-	3.73	3.53	4.00	-	-	-	-	26.55	-	-
Whisper-base (193 × 512)	LSTM-only	No	3.4M	0.6438	0.7660	0.5217	18	5	6	6	0.6686	0.7917	0.5455	19	4	6	6	0.5127	-	-
		Yes (*α* = 3e-5)	3.4M	0.6500	0.8000	0.5000	20	3	7	5	0.6749	0.8235	0.5263	21	2	7	5	0.6023	−1.7630	90.29
	*Δ* (in %)	-	-	0.96	4.44	−4.16	-	-	-	-	0.94	4.02	−3.52	-	-	-	-	17.48	-	-

TN = True Negative, FP = False Positive, FN = False Negative, TP = True Positive

**Table B. 2:** Results, in terms of F1-Score, Confusion-Matrix, $G_{V\;D}$ and DeID, for speaker disentanglement through LEV using the development set of the DAIC-WOZ dataset. The highlighted row (Δ) for each feature-model configuration indicates the relative change in the performance of that model without disentanglement versus our proposed method. The best F1-Score is bold-faced.

Input feature (Seq.len × Num. of features)	Model architecture	Speaker disentanglement	Model parameters	5-Models logit average							5-Models majority voting							F1-AVG (LLR)	*G_V D_* (in dB)	DeID in (%)
				F1-Score			Confusion Matrix				F1-Score			Confusion Matrix						
				F1(Avg)	F1(ND)	F1(D)	TN	FP	FN	TP	F1(Avg)	F1(ND)	F1(D)	TN	FP	FN	TP			
Mel-Spectrogram (120 × 40), (120 × 80)	CNN-LSTM	No	280k	0.6081	0.6977	0.5185	15	8	5	7	0.6578	0.7556	0.5600	17	6	5	7	0.6173	-	-
		Yes (*α* = 5e-5)	293k	0.6578	0.7556	0.5600	17	6	5	7	0.6830	0.7826	0.5833	18	5	5	7	0.5970	−0.1787	2.90
	*Δ* (in %)	-	-	8.17	8.30	8.00	-	-	-	-	3.83	3.57	4.16	-	-	-	-	−3.29	-	-
	ECAPA-TDNN	No	515k	0.6578	0.7556	0.5600	17	6	5	7	0.7086	0.8085	0.6087	19	4	5	7	0.5425	_-_	_-_
		Yes (*α* = 5e-2)	529k	0.6830	0.7826	0.5833	18	5	5	7	0.7464	0.8261	0.6667	19	4	4	8	0.5700	−0.5296	5.37
	*Δ* (in %)	-	-	3.83	3.57	4.16	-	-	-	-	5.33	2.18	9.53	-	-	-	-	5.07	-	-
Raw-Audio (61 440 × 1)	CNN-LSTM	No	445k	0.6259	0.7755	0.4762	19	4	7	5	0.6686	0.7917	0.5455	19	4	6	6	0.6182	_-_	_-_
		Yes (*α* = 1e-3)	459k	0.6686	0.7917	0.5455	19	4	6	6	0.7348	0.8333	0.6364	20	3	5	7	0.6429	−1.3189	61.98
	*Δ* (in %)	-	-	6.82	2.09	14.55	-	-	-	-	9.90	5.25	16.66	-	-	-	-	4.00	-	-
	ECAPA-TDNN	No	595k	0.6196	0.7391	0.5000	17	6	6	6	0.6941	0.7727	0.6154	17	6	4	8	0.5949	-	-
		Yes (*α* = 3e-3)	609k	0.7086	0.8085	0.6087	19	4	5	7	0.7348	0.8333	0.6364	20	3	5	7	0.6939	−0.5953	24.06
	*Δ* (in %)	-	-	14.36	9.39	21.74	-	-	-	-	5.86	7.84	3.41	-	-	-	-	16.64	-	-
ComparE16 (384 × 130)	CNN-LSTM	No	1.15M	0.5791	0.7234	0.4348	17	6	7	5	0.6941	0.7727	0.6154	17	6	4	8	0.4804	-	-
		Yes (*α* = 2e-4)	1.18M	0.6830	0.7826	0.5833	18	5	5	7	0.7552	0.8182	0.6923	18	5	3	9	0.4804	−0.0551	79.62
	*Δ* (in %)	-	-	17.94	8.18	34.15	-	-	-	-	8.80	5.89	12.50	-	-	-	-	0.00	-	-
Wav2Vec2.0-base (200 × 768)	LSTM-only	No	3.6M	0.6830	0.7826	0.5833	18	5	5	7	0.6830	0.7826	0.5833	18	5	5	7	0.5333	_-_	_-_
		Yes (*α* = 5e-3)	3.7M	0.6939	0.8163	0.5714	20	3	6	6	**0.7619**	0.8517	0.6667	21	2	5	7	0.6578	−0.3126	30.41
	*Δ* (in %)	-	-	1.60	4.31	−2.04	-	-	-	-	11.55	8.83	14.30	-	-	-	-	23.35	-	-
ContentVec-100 (193 × 768)	LSTM-only	No	3.6M	0.7287	0.7907	0.6667	17	6	3	9	0.7287	0.7907	0.6667	17	6	3	9	0.4804	-	-
		Yes (*α* = 2e-2)	3.7M	0.7287	0.7907	0.6667	17	6	3	9	0.7351	0.7805	0.6897	16	7	2	10	0.4804	−0.1416	18.50
	*Δ* (in %)	-	-	0.00	0.00	0.00	-	-	-	-	0.88	−1.29	3.45	-	-	-	-	0.00	-	-
WavLM-base (193 × 768)	LSTM-only	No	3.6M	0.6429	0.7143	0.5714	15	8	4	8	0.6941	0.7727	0.6154	17	6	4	8	0.5333	-	-
		Yes (*α* = 2e-3)	3.7M	0.6939	0.8163	0.5714	20	3	6	6	0.7200	0.8400	0.6000	21	2	6	6	0.5139	1.5854	72.71
	*Δ* (in %)	-	-	7.93	14.28	0.00	-	-	-	-	3.73	8.71	−2.50	-	-	-	-	−3.64	-	-
Whisper-base (193 × 512)	LSTM-only	No	3.4M	0.6438	0.7660	0.5217	18	5	6	6	0.6686	0.7917	0.5455	19	4	6	6	0.5127	-	-
		Yes (*α* = 5e-3)	3.4M	0.6830	0.7826	0.5833	18	5	5	7	0.6939	0.8163	0.5714	20	3	6	6	0.5127	−2.8950	84.49
	*Δ* (in %)	-	-	6.09	2.17	11.81	-	-	-	-	3.78	3.11	4.75	-	-	-	-	0.00	-	-

TN = True Negative, FP = False Positive, FN = False Negative, TP = True Positive

**Table B. 3:** Results, in terms of F1-Score, Confusion-Matrix, $G_{V\;D}$ and DeID, for speaker disentanglement through LECE using the development set of the DAIC-WOZ dataset. The highlighted row (Δ) for each feature-model configuration indicates the relative change in the performance of that model without disentanglement versus our proposed method. The best F1-Score is bold-faced.

Input feature (Seq.len × Num. of features)	Model architecture	Speaker disentanglement	Model parameters	5-Models logit average							5-Models majority voting							F1-AVG (LLR)	*G_V D_* (in dB)	DeID in (%)
				F1-Score			Confusion matrix				F1-Score			Confusion matrix						
				F1 (Avg)	F1 (ND)	F1 (D)	TN	FP	FN	TP	F1 (Avg)	F1 (ND)	F1 (D)	TN	FP	FN	TP			
Mel-Spectrogram (120 × 40), (120 × 80)	CNN-LSTM	No	280k	0.6081	0.6977	0.5185	15	8	5	7	0.6578	0.7556	0.5600	17	6	5	7	0.6173	-	-
		Yes (*α* = 4e-1)	293k	0.6684	0.7442	0.5926	16	7	4	8	0.6684	0.7442	0.5926	16	7	4	8	0.5238	−3.5427	72.13
	*Δ* (in %)	-	-	9.91	6.66	14.29	-	-	-	-	1.61	−1.51	5.82	-	-	-	-	−15.15	-	-
	ECAPA-TDNN	No	515k	0.6578	0.7556	0.5600	17	6	5	7	0.7086	0.8085	0.6087	19	4	5	7	0.5425	-	-
		Yes (*α* = 2e-7)	529k	0.6830	0.7826	0.5833	18	5	5	7	0.7464	0.8261	0.6667	19	4	4	8	0.5700	−0.489	5.91
	*Δ* (in %)	-	-	3.83	3.57	4.17	-	-	-	-	5.33	2.18	9.52	-	-	-	-	5.07	-	-
Raw-Audio (61 440 × 1)	CNN-LSTM	No	445k	0.6259	0.7755	0.4762	19	4	7	5	0.6686	0.7917	0.5455	19	4	6	6	0.6182	-	-
		Yes (*α* = 4e-5)	459k	0.7086	0.8085	0.6087	19	4	5	7	0.7086	0.8085	0.6087	19	4	5	7	0.6684	−0.5476	53.56
	*Δ* (in %)	-	-	13.21	4.26	27.82	-	-	-	-	5.98	2.12	11.58	-	-	-	-	8.12	-	-
	ECAPA-TDNN	No	595k	0.6196	0.7391	0.5000	17	6	6	6	0.6941	0.7727	0.6154	17	6	4	8	0.5949	-	-
		Yes (*α* = 3e-5)	609k	0.7348	0.8333	0.6364	20	3	5	7	0.7734	0.8511	0.6957	20	3	4	8	0.5333	−0.0446	15.62
	*Δ* (in %)	-	-	18.60	12.75	27.27	-	-	-	-	11.42	10.14	13.04	-	-	-	-	−10.35	-	-
ComparE16 (384 × 130)	CNN-LSTM	No	1.15M	0.5791	0.7234	0.4348	17	6	7	5	0.6941	0.7727	0.6154	17	6	4	8	0.4804	-	-
		Yes (*α* = 1e-7)	1.18M	0.5800	0.7600	0.4000	19	4	8	4	**0.8011**	0.8750	0.7273	21	2	4	8	0.4804	−1.0668	85.10
	*Δ* (in %)	-	-	0.16	5.06	−8.00	-	-	-	-	15.42	13.24	18.18	-	-	-	-	0.00	-	-
Wav2Vec2.0-base (200 × 768)	LSTM-only	No	3.6M	0.6830	0.7826	0.5833	18	5	5	7	0.6830	0.7826	0.5833	18	5	5	7	0.5333	-	-
		Yes (*α* = 5e-5)	3.7M	0.7619	0.8571	0.6667	21	2	5	7	0.7619	0.8571	0.6667	21	2	5	7	0.6939	−2.6701	63.55
	*Δ* (in %)	-	-	11.55	9.53	14.29	-	-	-	-	11.55	9.53	14.29	-	-	-	-	30.11	-	-
ContentVec-100 (193 × 768)	LSTM-only	No	3.6M	0.7287	0.7907	0.6667	17	6	3	9	0.7287	0.7907	0.6667	17	6	3	9	0.4804	-	-
		Yes (*α* = 5e-4)	3.7M	0.7552	0.8182	0.6923	18	5	3	9	0.7464	0.8261	0.6667	19	4	4	8	0.7086	−1.6775	59.11
	*Δ* (in %)	-	-	3.64	3.48	3.84	-	-	-	-	2.43	4.48	0.00	-	-	-	-	47.50	-	-
WavLM-base (193 × 768)	LSTM-only	No	3.6M	0.6429	0.7143	0.5714	15	8	4	8	0.6941	0.7727	0.6154	17	6	4	8	0.5333	-	-
		Yes (*α* = 2e-2)	3.7M	0.7472	0.8627	0.6316	22	1	6	6	0.7756	0.8846	0.6667	23	0	6	6	0.6686	−0.5155	76.98
	*Δ* (in %)	-	-	16.22	20.78	10.53	-	-	-	-	11.75	14.48	8.33	-	-	-	-	25.37	-	-
hisper-base (193 × 512)	LSTM-only	No	3.4M	0.6438	0.7660	0.5217	18	5	6	6	0.6686	0.7917	0.5455	19	4	6	6	0.5127	-	-
		Yes (*α* = 5e-6)	3.4M	0.6684	0.7442	0.5926	16	7	4	8	0.7552	0.8182	0.6923	18	5	3	9	0.3966	−3.7670	86.09
	*Δ* (in %)	-	-	3.82	−2.85	13.59	-	-	-	-	12.96	3.34	26.91	-	-	-	-	−22.64	-	-

TN = True Negative, FP = False Positive, FN = False Negative, TP = True Positive

**Table B. 4:** Results, in terms of F1-Score, Confusion-Matrix, $G_{V\;D}$ and DeID, for speaker disentanglement through LEKLD using the development set of the DAIC-WOZ dataset. The highlighted row (Δ) for each feature-model configuration indicates the relative change in performance of that model without disentanglement versus our proposed method. The best F1-Score is bold-faced.

Input feature (Seq.len × Num. of features)	Model architecture	Speaker disentanglement	Model parameters	5-Models logit average							5-Models majority voting							F1-AVG (LLR)	*G_V D_* (in dB)	DeID in (%)
				F1-Score			Confusion matrix				F1-Score			Confusion matrix						
				F1(Avg)	F1(ND)	F1(D)	TN	FP	FN	TP	F1(Avg)	F1(ND)	F1(D)	TN	FP	FN	TP			
Mel-Spectrogram (120 × 40), (120 × 80)	CNN-LSTM	No	280k	0.6081	0.6977	0.5185	15	8	5	7	0.6578	0.7556	0.5600	17	6	5	7	0.6173	-	-
		Yes (*α* = 5e-5)	293k	0.6578	0.7556	0.5600	17	6	5	7	0.6578	0.7556	0.5600	17	6	5	7	0.5970	−0.3079	11.42
	*Δ* (in %)	-	-	8.17	8.30	8.00	-	-	-	-	0.00	0.00	0.00	-	-	-	-	−3.29	-	-
	ECAPA-TDNN	No	515k	0.6578	0.7556	0.5600	17	6	5	7	0.7086	0.8085	0.6087	19	4	5	7	0.5425	-	-
		Yes (*α* = 1e-1)	529k	0.6941	0.7727	0.6154	17	6	4	8	0.7464	0.8261	0.6667	19	4	4	8	0.4853	−1.1953	1.90
	*Δ* (in %)	-	-	5.52	2.26	9.89	-	-	-	-	5.33	2.18	9.53	-	-	-	-	−10.54	-	-
Raw-Audio (61 440 × 1)	CNN-LSTM	No	445k	0.6259	0.7755	0.4762	19	4	7	5	0.6686	0.7917	0.5455	19	4	6	6	0.6182	-	-
		Yes (*α* = 2e-3)	459k	0.6830	0.7826	0.5833	18	5	5	7	0.7348	0.8333	0.6364	20	3	5	7	0.6684	−0.5503	37.05
	*Δ* (in %)	-	-	9.12	0.92	22.49	-	-	-	-	9.90	5.25	16.66	-	-	-	-	8.12	-	-
	ECAPA-TDNN	No	595k	0.6196	0.7391	0.5000	17	6	6	6	0.6941	0.7727	0.6154	17	6	4	8	0.5949	-	-
		Yes (*α* = 5e-3)	609k	0.7348	0.8333	0.6364	20	3	5	7	0.7348	0.8333	0.6364	20	3	5	7	0.6261	−2.2619	29.56
	*Δ* (in %)	-	-	18.59	12.75	27.28	-	-	-	-	5.86	7.84	3.41	-	-	-	-	5.24	-	-
ComparE16 (384 × 130)	CNN-LSTM	No	1.15M	0.5791	0.7234	0.4348	17	6	7	5	0.6941	0.7727	0.6154	17	6	4	8	0.4804	-	-
		Yes (*α* = 1e-2)	1.18M	0.6173	0.6829	0.5517	14	9	4	8	0.7287	0.7907	0.6667	17	6	3	9	0.4804	−4.6687	62.68
	*Δ* (in %)	-	-	6.60	−5.60	26.89	-	-	-	-	4.98	2.33	8.34	-	-	-	-	0.00	-	-
Wav2Vec2.0-base (200 × 768)	LSTM-only	No	3.6M	0.6830	0.7826	0.5833	18	5	5	7	0.6830	0.7826	0.5833	18	5	5	7	0.5333	-	-
		Yes (*α* = 5e-4)	3.7M	0.7009	0.8462	0.5556	22	1	7	5	0.7472	0.8627	0.6316	22	1	6	6	0.5333	−4.5179	55.83
	*Δ* (in %)	-	-	2.62	8.13	−4.75	-	-	-	-	9.40	10.24	8.28	-	-	-	-	0.00	-	-
Contentvec-100 (193 × 768)	LSTM-only	No	3.6M	0.7287	0.7907	0.6667	17	6	3	9	0.7287	0.7907	0.6667	17	6	3	9	0.4804	-	-
		Yes (*α* = 5e-4)	3.7M	0.7287	0.7907	0.6667	17	6	3	9	0.7351	0.7805	0.6897	16	7	2	10	0.4804	−0.1199	24.40
	*Δ* (in %)	-	-	0.00	0.00	0.00	-	-	-	-	0.88	−1.29	3.45	-	-	-	-	0.00	-	-
WavLM-base (193 × 768)	LSTM-only	No	3.6M	0.6429	0.7143	0.5714	15	8	4	8	0.6941	0.7727	0.6154	17	6	4	8	0.5333	-	-
		Yes (*α* = 5e-1)	3.7M	0.7086	0.8085	0.6087	19	4	5	7	0.7348	0.8333	0.6364	20	3	5	7	0.5139	0.9268	75.49
	*Δ* (in %)	-	-	10.22	13.19	6.53	-	-	-	-	5.86	7.84	3.41	-	-	-	-	−3.64	-	-
Whisper-base (193 × 512)	LSTM-only	No	3.4M	0.6438	0.7660	0.5217	18	5	6	6	0.6686	0.7917	0.5455	19	4	6	6	0.5127	-	-
		Yes (*α* = 1e-5)	3.4M	0.6830	0.7826	0.5833	18	5	5	7	0.7900	0.8800	0.7000	22	1	5	7	0.5139	−3.9297	69.42
	*Δ* (in %)	-	-	6.09	2.17	11.81	-	-	-	-	18.16	11.15	28.32	-	-	-	-	0.23	-	-

TN = True Negative, FP = False Positive, FN = False Negative, TP = True Positive.
